# Bayesian estimation of allele-specific expression in the presence of phasing uncertainty

**DOI:** 10.1093/bioinformatics/btaf283

**Published:** 2025-05-06

**Authors:** Xue Zou, Zachary W Gomez, Timothy E Reddy, Andrew S Allen, William H Majoros

**Affiliations:** Duke Center for Statistical Genetics and Genomics, Duke University, Durham, NC 27710, United States; Department of Biostatistics & Bioinformatics, Duke University Medical School, Durham, NC 27710, United States; Program in Computational Biology & Bioinformatics, Duke University, Durham, NC 27710, United States; Independent Researcher, Cambridge, Massachusetts, 02139, United States; Duke Center for Statistical Genetics and Genomics, Duke University, Durham, NC 27710, United States; Department of Biostatistics & Bioinformatics, Duke University Medical School, Durham, NC 27710, United States; Program in Computational Biology & Bioinformatics, Duke University, Durham, NC 27710, United States; Duke Center for Statistical Genetics and Genomics, Duke University, Durham, NC 27710, United States; Department of Biostatistics & Bioinformatics, Duke University Medical School, Durham, NC 27710, United States; Program in Computational Biology & Bioinformatics, Duke University, Durham, NC 27710, United States; Duke Center for Statistical Genetics and Genomics, Duke University, Durham, NC 27710, United States; Department of Biostatistics & Bioinformatics, Duke University Medical School, Durham, NC 27710, United States; Program in Computational Biology & Bioinformatics, Duke University, Durham, NC 27710, United States

## Abstract

**Motivation:**

Allele-specific expression (ASE) analyses aim to detect imbalanced expression of maternal versus paternal copies of an autosomal gene. Such allelic imbalance can result from a variety of cis-acting causes, including disruptive mutations within one copy of a gene that impact the stability of transcripts, as well as regulatory variants outside the gene that impact transcription initiation. Current methods for ASE estimation suffer from a number of shortcomings, such as relying on only one variant within a gene, assuming perfect phasing information across multiple variants within a gene, or failing to account for alignment biases and possible genotyping errors.

**Results:**

We developed BEASTIE, a Bayesian hierarchical model designed for precise ASE quantification at the gene level, based on given genotypes and RNA-Seq data. BEASTIE addresses the complexities of allelic mapping bias, genotyping error, and phasing errors by incorporating empirical phasing error rates derived from Genome-in-a-Bottle individual NA12878. BEASTIE surpasses existing methods in accuracy, especially in scenarios with high phasing errors. This improvement is critical for identifying rare genetic variants often obscured by such errors. Through rigorous validation on simulated data and application to real data from the 1000 Genomes Project, we establish the robustness of BEASTIE. These findings underscore the value of BEASTIE in revealing patterns of ASE across gene sets and pathways.

**Availability and implementation:**

The software is freely available from Github (https://github.com/x811zou/BEASTIE); and Zendo (DOI: 10.5281/zenodo.15062124).

## 1 Introduction 

Noncoding genetic variation has a major role in human traits and disease. For common and complex traits and diseases, genetic association studies typically identify non-coding genetic variants. Those associated variants have often been linked to impacts on gene regulatory activity (Alsheikh *et al.* 2022); and the narrow sense genetic heritability of such common traits and diseases is also strongly enriched in gene regulatory regions (Gusev *et al.* 2014). Non-coding genetic variation also plays a major role in rare diseases that collectively impact an estimated 3%–4% of the global population (Amberger *et al.* 2015; Frésard *et al.* 2019). For example, the diagnostic yield of rare diseases via DNA sequencing is 50% (Boycott *et al.* 2017). Supplementing DNA sequencing with gene expression measurements and RNA sequencing provides additional information about the potential disruption of a gene in a patient. In some cases, doing so has increased diagnostic rates by upwards of 15% through analysis of altered gene splicing, allele-specific expression, and unusual gene expression levels (Peymani *et al.* 2022). Together, these findings suggest that including gene expression measurements in studying traits and diseases can substantially improve our understanding of disease mechanisms.

Allele-specific expression (ASE), defined as an allelic imbalance in transcript quantity between maternal and paternal copies of autosomal genes, is an important indicator of a possible gene expression outlier. Altered gene expression can arise via many mechanisms including genetic variation in promoters and enhancers and loss-of-function mutations that impact mRNA splicing or stability and result in transcript degradation. Predicting such effects from DNA sequencing alone remains an unmet challenge. Compared to differential gene expression analysis, ASE analysis also has the advantage that it compares the expression of copies of a gene within the same nucleus, potentially increasing the power to detect expression anomalies. Recent advancements have illustrated the utility of incorporating ASE analysis in rare disease diagnostics, for example, elevating the diagnostic rate by 7.5% across various disease types (Cummings *et al.* 2017; Kremer *et al.* 2017; Frésard *et al.* 2019). Further, ASE analysis stands as a pivotal tool for identifying the existence of potential cis-regulatory effects in gene expression in both human and non-human organisms (Heap *et al.* 2010; Cummings *et al.* 2017; Li *et al.* 2017; Llavona *et al.* 2017; Stachowiak *et al.* 2018; Frésard *et al.* 2019).

The estimation of ASE can be strongly confounded by a number of issues, including (1) the availability of exonic heterozygous sites in a gene; (2) the need to combine read counts across sites in a phase-consistent manner; (3) the possibility of genotyping errors; and (4) allelic mapping bias. We discuss these in detail below.

*Availability of heterozygous sites*. Among 445 individuals from the 1000 Genome Project, the average number of heterozygous (het) sites per gene is 3.3. Roughly 30% of genes have one exonic het SNP, while 34% have 4 or more exonic het SNPs. While some methods make use of only one variant in a gene, others attempt to make use of all sites by aggregating read counts across sites (Skelly *et al.* 2011; Mayba *et al.* 2014). When using short-read sequencing, individual reads may span only a subset of those variants, which presents the problem of how to combine read counts across sites within a gene, as using the counts at only one site to estimate ASE fails to make use of all available information. This is the phasing problem: determining which alleles at multiple het sites originate from the same chromosome.

*Combining read counts across sites in a phase-consistent manner*. For short reads spanning individual het sites, allelic read counts need to be combined across sites in a manner that observes phasing, so that reads are summed across the maternal and paternal chromosomes separately. Unfortunately, the relative phasing of sites is often not known with certainty. One class of methods heuristically addresses this problem by utilizing pseudo-phasing (Mayba *et al.* 2014; Fan *et al.* 2020), which assumes that the alleles with the higher read count at each site always originate from the same gene copy. While there should be a statistical tendency for the alleles with higher counts to often come from the same gene copy in the presence of ASE, the assumption that they always do so is not generally correct, particularly under the null hypothesis (no ASE), and thus pseudo-phasing can result in overestimation and elevated Type I error. A more principled approach is to either predict a single best-supported phasing and use that as a proxy for the true phasing or to explicitly account for phasing uncertainty statistically when estimating ASE. Using a single predicted phasing [e.g. from methods such as phASER (Castel *et al.* 2016) or SHAPEIT2 (O'Connell *et al.* 2014)] has the shortcoming that the ASE estimate will be impacted by any errors in the predicted phasing. As we describe shortly, our approach instead explicitly accounts for phasing error rates when estimating the full posterior distribution of ASE, while marginalizing out the true phasing in a statistically rigorous way.

*Genotyping error*. Genotyping errors occur when variant calling or genotyping algorithms mislabel genotypes, leading to a zero read count on an allele in RNA (Rivas *et al.* 2015) and false signals of allelic imbalance. Various approaches have been proposed to remove sites with incorrect genotypes, including the use of customized genotype error filters that consider sequencing noise and coverage (Castel *et al.* 2015), developing a customized genotype caller to compute genotype at each SNP position (Romanel *et al.* 2015), deploying a new genotype caller that only requires RNA-Seq data (Deonovic *et al.* 2017), jointly inferring genotypes in a ASE model (Harvey *et al.* 2015), and filtering nonsense variants if the alternative allele ratio was low (Kukurba *et al.* 2014). Some researchers have instead relied on having higher-quality source data (Degner *et al.* 2009; Skelly *et al.* 2011; Lappalainen *et al.* 2013; Castel *et al.* 2015; Kukurba and Montgomery 2015; Knowles *et al.* 2017; Li *et al.* 2017), such as HapMap, 1000 Genomes, and GIAB, without additional base-calling correction, thereby constraining their analyses to data with established low base-calling errors.

*Allelic mapping bias*. Allelic mapping bias arises when reads that contain the reference allele at a heterozygous site are successfully aligned to the reference genome at a higher rate than reads bearing the alternate allele, leading to skewed expression estimates that do not accurately reflect the true allelic expression ratios (Kukurba *et al.* 2014). To address this challenge, prior studies have adopted various methods such as using variant-aware aligners (Castel *et al.* 2015; Hodgkinson *et al.* 2016; Garrison *et al.* 2018), implementing adjusted binomial models to take into account the pre-existing allelic bias (Mayba *et al.* 2014; Romanel *et al.* 2015), and filtering out sites through customized mappability filters or simulations (Skelly *et al.* 2011; Lappalainen *et al.* 2013; Castel *et al.* 2015; Harvey *et al.* 2015; Van De Geijn *et al.* 2015; Li *et al.* 2017).

Here we introduce BEASTIE, a novel approach for estimating ASE that accounts for all of the foregoing challenges, in a statistically principled way. By leveraging a probabilistic Bayesian framework, this model allows for the incorporation of prior information to yield more precise posterior estimates of allelic imbalance. BEASTIE makes use of an external phasing algorithm but accounts for possible phasing errors in a locus-specific and variant-specific manner by studying local phasing error rates and using those to statistically marginalize over all possible phasings when estimating ASE. Doing so enables BEASTIE to produce more robust estimates than methods that either use only one site or assume a single predicted phasing without accounting for possible phasing errors. In addition to its ability to include alignment bias filtering and genotyping error filtering—features that many existing methods lack (Table 1, available as supplementary data at *Bioinformatics* online)—We also provide a processing pipeline that mitigates alignment bias by simulation and streamlines the processing of raw RNA-Seq fastq reads and VCF data to compute and output gene-level ASE, establishing BEASTIE as a comprehensive tool for the investigation of allelic imbalance.

**Table 1.** Definitions of the variables in Fig. 1.

Variable	Definition
θ	Effect size (ASE) (latent)
$N_{i}$	Total read count at site *i*
$X_{i}$	Alternate allele read count at site *i*
$\pi_{i}$	Switching error rate between sites *i* and i+1
$\phi_{i}$	True phasing of site *i* (latent)
$\phi_{i}^{*}$	Predicted phasing of site *i*
*n*	Number of exonic heterozygous sites in gene
ϵ	Probability of transitioning to start/stop state
even	State representing an even number of switching errors
odd	State representing an odd number of switching errors

BEASTIE estimates ASE within individual genes using heterozygous SNPs from exonic regions. Although we only use variants from exonic regions to detect and quantify ASE, the underlying cause of the ASE that we measure may originate from regulatory elements located within or beyond the gene body, such as enhancers and promoters, or may originate within the gene itself.

## 2 Materials and methods

**Figure 1.**
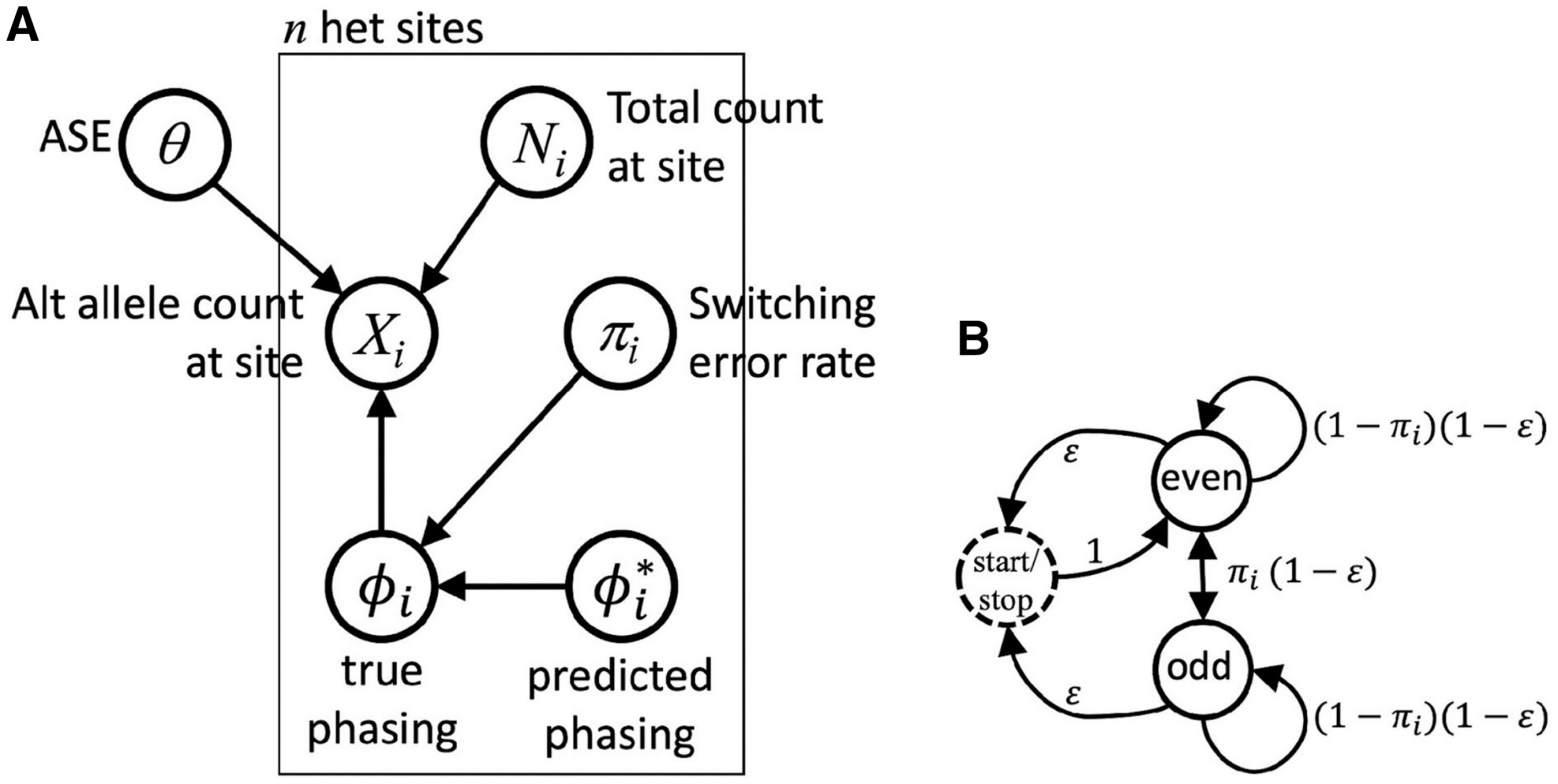
(A) The BEASTIE model for estimating ASE from multiple exonic heterozygous sites in a gene while accounting for phasing uncertainty. (B) An inhomogeneous hidden Markov model for summing in linear time over all possible phasings based on site-specific phasing error rates.

In order to integrate information across multiple heterozygous sites within a gene while accounting for phasing uncertainty, we designed a Bayesian probabilistic graphical model in which both ASE and phasing are treated as latent variables. Because these entities are both latent, we can do joint inference on them, thus allowing our estimate of ASE to take into account our uncertainty about phase. The resulting model, BEASTIE, is depicted in Fig. 1. Variable definitions are given in Table 1.

BEASTIE is intended to be applied to each gene independently to estimate the degree of ASE, expressed as an odds, θ:


$$\theta =\frac{p}{1-p}$$

where *p* is the relative proportion of expression of the gene originating from the maternal copy. Note that we arbitrarily label one chromosome as maternal, for simplicity, given that the two chromosomes are exchangeable under our statistical model.

Inputs to the model consist of *n*, the number of exonic heterozygous sites in the gene; $N_{i}$, the total number of reads spanning site *i*; $X_{i}$, the number of reads spanning site *i* that contain the alternate allele; $\pi_{i}$, the expected per-site switching error rate (for phasing) between sites *i* and i+1; and $\phi_{i}^{*}$, the predicted phasing of site *i*. Read counts $N_{i}$ and $X_{i}$ can be obtained by simple counting of reads aligned to the gene that spans each site; to avoid double-counting individual reads that span multiple sites, we provide a script to thin the list of heterozygous sites so that no two sites are within a read-length of each other in transcript distance.

The expected switching error rates $\pi_{i}$ are provided by a separate model, Switching Error Logistic Regressor (SELR), detailed in Text 2.3, available as supplementary data at *Bioinformatics* online, which predicts site-specific switching error rates based on linkage disequilibrium, allele frequencies, and distances between sites. We provide a pre-trained version of the model tuned specifically for the statistical phaser SHAPEIT2 [17]; a script is also provided for re-training the SELR model for arbitrary phasing programs. We also provide a version of BEASTIE that does not require site-specific error rate estimates. That version, called BEASTIE-latent, treats the phasing error rate as a latent variable, which is automatically marginalized out of the model during inference.

Due to the exchangeability of maternal versus paternal chromosome labels in our model, and with the relative nature of statistical phasing (i.e. statistical phasing of a given site is generally only meaningful in relation to the phasing of other sites), we model relative rather than absolute phasing. Thus it is sufficient to fix one site and phase all others relative to that site. To promote identifiability of the model, we choose the site with the highest coverage as the fixed site (details can be found in Fig. 1, available as supplementary data at *Bioinformatics* online); that is, we assume $\phi_{i}=\phi_{i}^{*}$ where *i* is the highest coverage site. Phasing at this site serves as a reference, with the model assessing phasing for other sites in the gene relative to that site.

Given this arbitrary phasing of the site with the highest coverage, the phasing of all other sites relative to that site determines how the binomial likelihoods are evaluated. In particular, the likelihood of $X_{i}$ is a binomial in which the probability parameter is either *p* (when the alternate allele is on the maternal chromosome) or 1−p (when the alternate allele is on the paternal chromosome). Our BEASTIE model has two implementations. For the STAN version (Stan Development Team, 2024), missing phasing error rates are treated as latent variables, as described in Text Section 2.1, available as supplementary data at *Bioinformatics* online. In the C++ version, phasing errors are incorporated as parameters determined by SELR or set as default parameters (5%), as outlined in Text Section 2.2, available as supplementary data at *Bioinformatics* online.

## 3 Results

### 3.1 Phasing ambiguity is an important problem in estimating ASE

**Figure 2.**
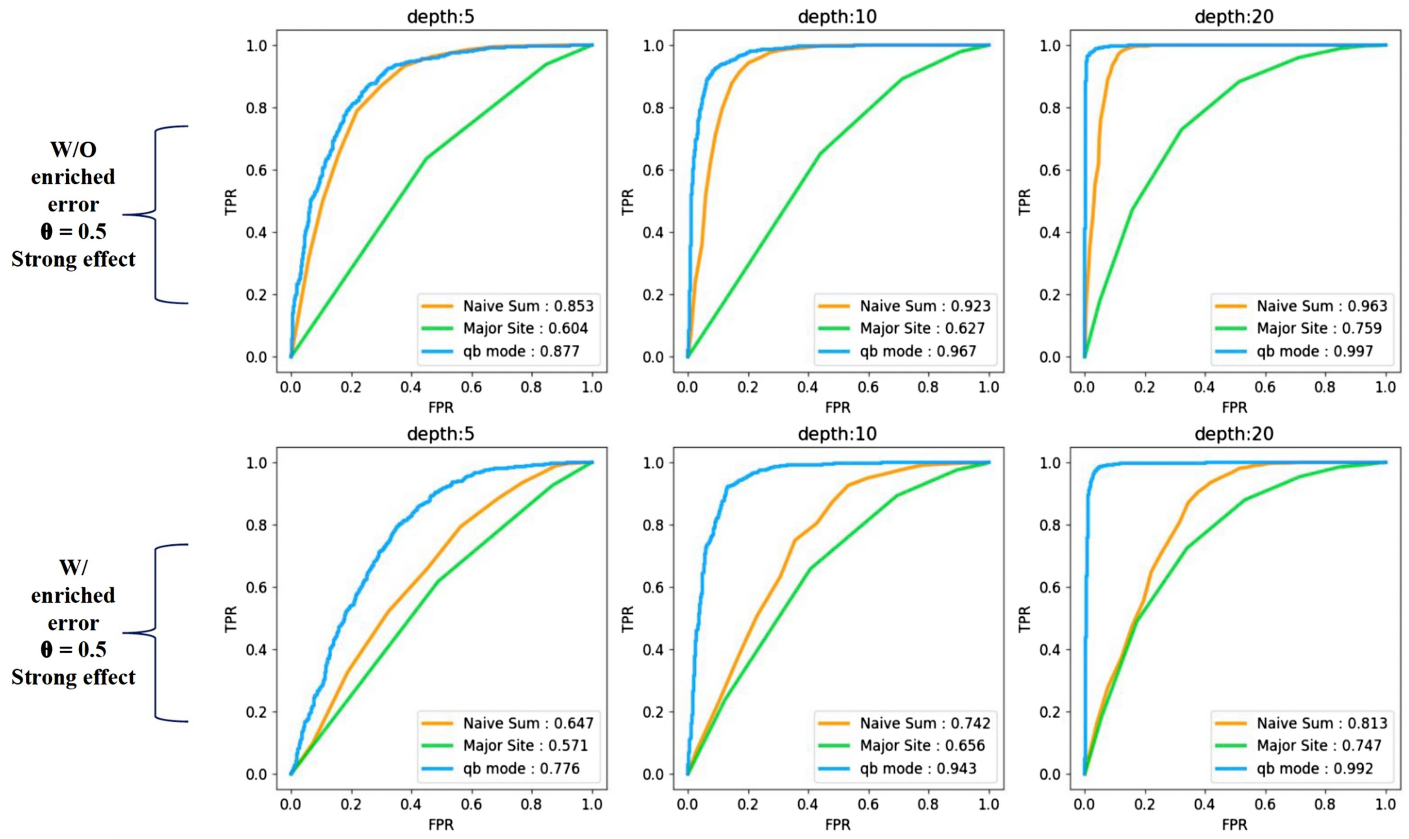
ROC curves for BEASTIE and baseline methods on simulated data. (Upper) ROC curves on simulated data from GIAB data with 3.8% switching error, 10 heterozygous sites per gene, and coverage of 5, 10, or 20 reads per site, with strong ASE effect (θ = 0.5). (Bottom) ROC curves on simulated data from GIAB data with 12% switching error (an odd number of switching errors per gene), 10 heterozygous sites per gene, and coverage of 5, 10, or 20 reads per site, with strong ASE effect (θ = 0.5). All panels: Legends show AUC values. Semi-empirical simulators composed 1000 genes with 10 heterozygous sites per gene from real GIAB data. ROC curves compare BEASTIE with predicted phasing error and two baseline methods. Results are from the optimized C++ BEASTIE (*qb mode*), described in Text Section 2.2, available as supplementary data at *Bioinformatics* online.

We conducted an analysis of the number of exonic heterozygous (het) sites within each gene across 445 individuals from the 1000 Genomes Project and observed a long-tailed distribution (Fig. 2, available as supplementary data at *Bioinformatics* online), with 34% of genes containing four or more exonic het sites, and an average of 3.3 exonic het sites per gene. This distribution highlights the need for analytical methods that aggregate information across multiple genetic loci. The limitations of short-read sequencing technologies, which often capture only fragments of the transcript sequence, necessitate accurate assembly of these fragments.

Accurate aggregation of read counts across sites requires resolving their phase. Even in trio data, in which we can use parental genetic information to phase the offspring’s het sites, triple heterozygous sites (triple-hets) remain unphased. In one trio from the 1000 Genomes Project (individuals NA19240, NA19238, and NA19239; Text 1.4, available as supplementary data at *Bioinformatics* online), approximately 20% of sites were unphaseable due to triple-het configurations. This was consistent with simulations using trio transmission data derived from parental samples of diverse ancestries (Table 2, available as supplementary data at *Bioinformatics* online).

We evaluated the phasing accuracy of SHAPEIT2, one of the most widely used statistical phasers, in three individuals. In NA19240, phased using trio phasing, we observed a mean 2.200% switching error rate for phasable child heterozygous SNP pairs. In addition, our analysis of individuals from two ancestries documented genome-wide switching error rates excludes cross-gene SNP pairs, finding an average rate of 4.030% for a Utah female (NA12878) against the GIAB gold standard reference phasing and average rate of 4.025% for a Yoruba male (GM19440), using experimentally determined haplotype phasing (Table 4, Supplementary Text 1.2 and 1.3, available as supplementary data at *Bioinformatics* online).

In NA12878, despite the average pairwise switching error rate being low (4.030%, 974 out of 24 167), we observed large variation in switching error rates across the genome. In particular, our study identified instances of high phasing errors particularly in genes with larger numbers of exonic het sites (Table 5, available as supplementary data at *Bioinformatics* online; Spearman ρ = 0.685, *p *= 0.002), SNP pairs separated by long distances, and SNPs with low minor allele frequencies (MAFs), as detailed below. We found a strong and statistically significant correlation between the number of exonic heterozygous sites within a gene and the likelihood of phasing errors. Our logistic regression analysis (error∼num.of.hets) revealed that as the number of heterozygous sites increases, the probability of encountering phasing errors also increases significantly (Fig. 3, available as supplementary data at *Bioinformatics* online). This relationship was confirmed by the ANOVA (code detailed in Text 4.3, available as supplementary data at *Bioinformatics* online), where the inclusion of the number of heterozygous sites as a predictor resulted in a substantial reduction in residual deviance (from 1734.2 to 1718.1) and an extremely low *P*-value (5.897e-05), indicating a highly significant effect. We also noted that the gene level switching error rate, i.e., the rate at which you see at least one switching error in a gene, increases substantially with the number of hets in a gene. Specifically, we noted a percentage of 6.190% of genes (out of total 9616 genes with 2 hets) having at least one switching error, increasing to 7.425% for three hets, and peaking at 30.556% for genes with thirteen hets (with each category having at least 10 genes) (Table 5, available as supplementary data at *Bioinformatics* online). Examination of the MAF groups for each SNP pair demonstrated a contrast in average phasing error rates based on allele rarity (Fig. 4B, available as supplementary data at *Bioinformatics* online), with rare SNPs (MAF < 0.2%) exhibiting average phasing error rates as high as 48.55%, whereas common SNPs (MAF > 5%) had significantly lower average phasing error rates, at 3.12% (Table 6, available as supplementary data at *Bioinformatics* online). Furthermore, phasing error rates increased significantly from 4% to 8.37% when inter-SNP distances exceeded 1 kilobase, emphasizing the challenges in accurately phasing variants over larger genomic distances.

### 3.2 BEASTIE performance

#### 3.2.1 BEASTIE outperforms competing methods on realistic simulation data

We evaluated the performance of BEASTIE in comparison to two established methods: MajorSite (MS) and NaiveSum (NS), which build upon the methodologies reported in earlier studies (Jing *et al.* 2011; Castel *et al.* 2015; Castel *et al.* 2016). MajorSite makes use of only one site within each gene—the site with the highest count. When multiple sites are present in a gene, MajorSite fails to make use of all available information. On the other hand, NaiveSum (NS) aggregates allelic read counts across exonic heterozygous sites within a gene, under the assumption of perfect phasing and drawing on concepts from previous studies (Romanel *et al.* 2015). However, the accuracy of this method is compromised by the existence of phasing errors, resulting in the summation of incorrect counts. A third approach is pseudo phasing, which assumes that the allele with the higher count at each site originates from the same haplotype, an approach introduced by MBASED (Mayba *et al.* 2014). This method is excluded from our comparison due to its inability to adequately control type 1 error rate.

This comparative analysis focused on each method’s proficiency in distinguishing positive cases (nonzero ASE) from negative cases (zero ASE) within simulated datasets having 10 het sites per gene. We selected genes with 10 het sites to highlight BEASTIE’s ability to detect true ASE signals despite high phasing error, which is challenging for existing methods. Fixing the number of heterozygous sites while varying read depth allowed us to observe a clearer and more pronounced difference in model performance. We also evaluated model performance on genes with 1–6 heterozygous sites at a fixed read depth of 10, as shown in Fig. 6, available as supplementary data at *Bioinformatics* online. These additional evaluations demonstrate that the advantage of BEASTIE over baseline methods remains consistent even with fewer heterozygous sites. We simulated read depths ranging from 5 to 20 reads per variant and effect sizes of θ = 0.5 or 0.75. The discriminative efficacy was quantified via AUC values (details in Text 1.6, available as supplementary data at *Bioinformatics* online), particularly in distinguishing genes with θ = 0.5 or 0.75 from genes conforming to the null hypothesis (θ=1).

Empirical findings demonstrated NaiveSum’s superiority over MajorSite at 5% or 10% switching error rates, demonstrating the utility of combining information across multiple sites. In scenarios devoid of switching errors, NaiveSum’s performance was on par with BEASTIE, while MajorSite was disadvantaged in multi-site contexts. Notably, BEASTIE outperformed NaiveSum in datasets exhibiting non-zero switching error rates (Fig. 6A, available as supplementary data at *Bioinformatics* online).

For genes with 10 heterozygous sites, the accuracy of BEASTIE with default 5% phasing error was predominantly influenced by the sequencing depth per site and the effect size. An increase in read depth from 5 to 20 reads per site substantially enhanced BEASTIE’s accuracy (AUC from 0.898 to 0.998), especially when the switching error rate was 5% and the effect size was 0.5.

Elevating the switching error rate to 10% resulted in a diminished accuracy at lower read counts; however, at a simulated 20 reads per site, the accuracy of BEASTIE remained stable (AUC between 0.994 and 0.998) for data with switching error rates ranging from 0–10% (Fig. 6B, available as supplementary data at *Bioinformatics* online). Furthermore, a decrease in the effect size from θ = 0.5 to θ = 0.75 significantly reduced BEASTIE’s accuracy (AUC from 0.827 to 0.747) in datasets with 20 reads per site and switching error rates between 0–10%. While a depth of 5 reads per site was insufficient for reliable detection of subtler effects, enhancing the depth to 20 reads per site significantly improved accuracy (AUC at 0.747 for θ = 0.75), even in scenarios characterized by weaker effects and a 10% switching error rate (Fig. 6C, available as supplementary data at *Bioinformatics* online).

#### 3.2.2 Phasing error rates are accurately predicted by genetic and genomic features

To enhance the accuracy of phasing error estimates within the BEASTIE framework, we developed the Switching Error Logistic Regressor (SELR), a logistic regression model designed to predict the switching error rate of SNP pairs processed by SHAPEIT2. This model was calibrated on data from the NA12878 sample, and SHAPEIT2 phasing errors were identified by comparing each SNP pair’s phasing against the gold standard phasing (from GIAB), with discrepancies marked as errors.

SELR utilizes a number of predictive features, including the minimum minor allele frequency (MAF) within the SNP pair, the differential MAF between SNP pairs, the ${\;\text{log}\;}_{10}$-transformed inter-SNP distance, and the linkage disequilibrium (LD) metrics ($r^{2}$ and $D^{\prime }$), as well as second-order interaction terms between these variables (Table 8, available as supplementary data at *Bioinformatics* online). GIAB data were randomly split into two halves: the model was trained on 8729 site pairs and validated against a disjoint set of 8730 site pairs, each set comprising ∼3.7% incorrectly phased site pairs. SELR attained an impressive AUC of 0.8425 on validation data set (Fig. 7B, available as supplementary data at *Bioinformatics* online), indicating a high level of discrimination between correctly and incorrectly phased site pairs. These features were selected based on their observed correlations with switching error rates, and the training/testing details as described in Text 2.3, available as supplementary data at *Bioinformatics* online.

Our analysis on NA12878 data identified several trends: average SHAPEIT2 phasing error rates increase with greater inter-SNP distances and decrease in scenarios of high MAF and strong LD ($r^{2}$ and $D^{\prime }$). Similar trends were observed in the predicted switching error rate per SNP pair from SELR (Figs 4 and 5, available as supplementary data at *Bioinformatics* online). These findings align with the expectation that for statistical phasing methods such as SHAPEIT2, rarer SNPs and those with greater distances between them are more prone to switching errors, as are SNP pairs characterized by low LD. Additionally, we observed a consistent trend across all chromosomes where higher MAF values correlate with reduced switching errors, while rare SNPs, particularly those with less than 0.2% MAF, are associated with increased switching errors (Figs 8–10 and Tables 6 and 7, available as supplementary data at *Bioinformatics* online). This pattern extends to LD values, where SNP pairs with lower LD are more likely to exhibit higher switching errors.

Upon integrating this model-predicted switching error rate into the BEASTIE framework, supplanting the fixed 5% phasing error parameter, we observed a notable performance enhancement (Fig. 2).

#### 3.2.3 Computational efficiency and scalability

Estimating ASE using Bayesian methods can be computationally intensive, particularly when generating null distributions for multiple hypothesis testing. In BEASTIE, calculating *P*-values from 10 000 null simulations per gene requires approximately 3 h for 8000 genes on a high-performance computing cluster using a single core of a 2.2 GHz Intel Xeon processor, making large-scale analysis impractical. To address this limitation, we developed QuickBEAST, a C++ implementation optimized for speed and parallelization. By leveraging dynamic programming and efficient Bayesian updating, QuickBEAST reduces the runtime from 3 h to 4 min for 8000 genes, enabling practical large-scale ASE analysis (Text 2.2, available as supplementary data at *Bioinformatics* online).

### 3.3 ASE traits on 1000 genome dataset

In this study, BEASTIE was applied to a dataset of 445 samples from the 1000 Genomes Project (1KGP), representing five distinct ancestries. This dataset encompassed high-throughput RNA-Seq data from lymphoblastoid cell lines for each individual, coupled with complete 1000 Genomes phase3 genotype data. Our primary aim was to investigate the ASE landscape across different ancestries and examine ASE signals from gene sets of interest. Notably, prior ASE studies employing 1000 Genome data have assumed perfect phasing and genotyping from the 1000 Genome phase 1 dataset and Omni 2.5M SNP array data (Delaneau and Marchini 2014; Tian *et al.* 2018). In contrast, our analyses are based on our BEASTIE method that accommodates the potential for phasing errors, and are based on 1000 Genomes phase 3 data (details can be found in Text 1.1, available as supplementary data at *Bioinformatics* online).

#### 3.3.1 BEASTIE identifies the most ASE genes

In our study analyzing this large cohort, we found that, per individual, an average of 7.23% (range: 3.14%–19%; Table 11, available as supplementary data at *Bioinformatics* online) of genes exhibit ASE. This was observed in genes possessing at least one het site and having on average at least 10 reads per site. In comparison, when employing Naive Sum and Major Site, the proportions of genes exhibiting ASE were identified to be 6.39% and 4.37%, respectively.

#### 3.3.2 ASE magnitude and enrichment between genesets

Our study analyzed ASE across twelve gene sets, including imprinted genes, genes tolerant to loss-of-function (LoF), genes associated with recessive conditions as per the Residual Variation Intolerance Score (RVIS), housekeeping genes, autosomal dominance (AD), and several others related to haploinsufficiency and essential functions, as delineated in Table 13, available as supplementary data at *Bioinformatics* online. We assessed ASE rates by calculating the ratio of ASE-positive genes within each gene set for individual samples. A comparison of ASE rates between specialized gene sets and a defined “non-specialized” set of genes (not belonging to any of the gene sets considered here) revealed significant differences (Wilcoxon sign-rank test P<0.05; Table 14, available as supplementary data at *Bioinformatics* online). Further analysis focused on ASE magnitudes ($\left|{\;\text{log}\;}_{2}(\theta )\right|$), revealing skewed distributions within each gene set, indicating diverse magnitudes of ASE in genes identified as having expression imbalance.

Known imprinted genes exhibited a notably high mean ASE magnitude of 3.49, reflecting strong epigenetic control, in contrast to housekeeping genes, which had a lower mean of 0.526, consistent with their role in maintaining cellular function. Genes with ASE showed a narrow mean ASE magnitude range (1.02–1.18) across genesets (LoF tolerant, recessive, OMIM haploinsufficient, AD) with all genes average to be 1.30 and non-specialized geneset to be 1.31 (Table 15, available as supplementary data at *Bioinformatics* online). Permutation tests comparing ASE magnitudes on ASE genes across genesets confirmed these observations without relying on distributional assumptions, using over 10 000 randomizations for robustness. Interestingly, genes without ASE showed a narrow mean ASE magnitude range (0.213–0.265) across 12 genesets (Table 16 and Fig. 14, available as supplementary data at *Bioinformatics* online).

The differentiation in ASE rates and magnitudes between genesets, particularly between imprinted and stillbirth genes, illustrates the complexity of patterns in gene expression imbalance in our dataset. Specifically, the housekeeping geneset, with a lower ASE rate of 21.6% (versus imprinted genes of 63.8%), emphasizes their stable expression critical for cellular homeostasis. The mean ASE rate of all genes is 6.89. Non-specialized geneset (7.00), LoF geneset (13.4), recessive geneset (5.98), ClinGen haploinsufficient geneset (3.97), OMIM haploinsufficient geneset (5.29), All haploinsufficient (4.02), MGI essential geneset (6.21), and AD geneset (6.47), stillbirth (1.64). The significant differences in ASE rates between genesets such as stillbirth genes, ClinGen haploinsufficiency, MGI essential genes, and the non-specialized set further highlight the complex interplay of developmental and disease processes (Tables 14, available as supplementary data at *Bioinformatics* online).

### 3.4 Genes with extreme ASE magnitude

We employed a gene selection strategy to identify both known and potential novel candidates for imprinted genes in LCL, successfully highlighting the top 25 genes that exhibit extreme ASE (significant FDR adjusted *P*-value & absolute value of log2 ASE estimates needs to be in the top quartile), as detailed in Table 17 and Fig. 22, available as supplementary data at *Bioinformatics* online. Our selection criteria identifies the top 25 genes with the highest percentage of individuals that show extreme ASE. We further restrict genes that show a consistent pattern of extreme ASE across 5 ancestries. In particular, we only consider genes where at least two individuals from each ancestry have extreme ASE. This final set includes 6 previously documented genes in specific tissues: NAPIL5, HLA-DQA2, FAM50B, LPAR6, UTS2, and ZDBF2 (Table 18, available as supplementary data at *Bioinformatics* online), along with two well-known imprinted genes in peripheral blood lymphocyte, PEG10 and SNRPN (Frost *et al.* 2010). The remaining 17 genes (Table 19, available as supplementary data at *Bioinformatics* online), which include 3 pseudogenes, 2 RNA genes, and 6 immune gene segments, lack prior literature on their imprinting status, necessitating further validation to confirm their extreme ASE profiles. The high allelic imbalance on immunoglobulin (Ig) gene loci might be caused by misalignment issues within the V(D)J recombination regions (Rizzetto *et al.* 2018). There is no expectation that the allele (reference versus alternate) would correlate with ASE status in imprinted genes, and indeed, no such association was observed (Fisher’s exact test I, all P>0.1; Table 20, available as supplementary data at *Bioinformatics* online).

## 4 Discussion

ASE analyses complement eQTL analyses by focusing on expression imbalance without assuming a specific mechanism. The internal control provided by comparing allelic counts within a single sample may offer stronger power from smaller sample sizes than eQTL analyses in certain cases. Furthermore, ASE analyses do not assume any particular mechanism for differences in allelic expression, making them flexible tools for exploring gene regulation.

Here we present BEASTIE, a novel Bayesian approach for detecting ASE that rigorously accounts for phasing uncertainty by treating phasing as a latent variable that is integrated out during inference. Previous solutions to estimating ASE rely on a single heterozygous site, a single predicted phasing, or pseudo phasing to combine read counts across sites. As we have shown, those strategies may not control false positive rates, are subject to incorrect phasing prediction, and ultimately miss opportunities to use all available phasing information. BEASTIE builds upon those previous studies by integrating information across exonic sites and incorporating additional information such as population allele frequencies, the distance between SNPs, and linkage disequilibrium. Through the BEASTIE modeling approach, we address several confounding challenges when estimating ASE. Specifically, we allow users to better assess the strength of evidence for allelic imbalance by reporting the full posterior distribution of ASE; we more precisely estimate phasing error rates; and we correct for systematic biases due to alignment errors and genotyping errors. Those features are particularly valuable for identifying issues such as multi-modality or highly dispersed posterior mass, which can inform the interpretation of ASE estimates.

We view this study as particularly impactful in two key applications of ASE analysis. First, there is often little phasing certainty for rare genetic variants, especially when they are more than 1 kb from a common variant. By integrating over that uncertainty, we can improve ASE inference involving such rare variants. That can potentially improve the use of RNA assays in diagnosing rare diseases and in understanding the effects of emerging variations in the human population. Second, there is also often more phasing uncertainty for genomes from populations and ancestries without established reference panels. Again, by integrating that potential uncertainty, we can improve ASE studies for more diverse populations. Both of these advances are important steps toward making genetics work for everyone.

BEASTIE’s ability to detect both known imprinted genes and genes with extreme ASE magnitudes underscores its utility in expanding our understanding of gene expression regulation. Our analysis of real data from the 1000 Genomes Project indicates that the distribution of ASE effect sizes (θ) range between 0.01 and 97 with many genes having a consistently large ASE effect. Specifically, we identified 25 additional genes with consistently extreme ASE across many individuals, including 2 genes known to be imprinted in blood and 6 with imprinting status on other tissues mentioned in prior publications. Among those, all exhibited no significant association between haplotype and gene expression. Six of those genes are immunoglobulins. For those, the extreme ASE is likely due to a combination of V(D)J recombination, jackpotting in lymphoblastoid cell cultures, and alignment artifacts. The remaining 11 genes may be novel candidates for imprinting in the human genome, or may instead be subject to random inactivation or genetic alteration via other mechanisms.

Our findings also reveal significant differences in ASE rates across various gene sets, reflecting the complex interplay between genetic regulation and functional categories. The observed higher ASE magnitudes in imprinted genes, compared to housekeeping genes, are consistent with strong epigenetic regulation in imprinted loci. In contrast, the narrow range of ASE across other gene sets indicates a degree of stability in expression imbalance that may be reflective of underlying genetic or regulatory constraints. The significant depletion of ASE in the stillbirth gene set in particular suggests stringent selective pressure to maintain stable expression in genes crucial for cellular homeostasis and organismal viability. These results align with the expected selection against disruption in essential genes, further illustrating the nuanced regulatory landscape that BEASTIE can help to elucidate.

A notable strength of BEASTIE is its generalizability to different phasing tools beyond SHAPEIT2. While our current implementation utilizes SHAPEIT2, the model is designed to accommodate other phasers such as Eagle and Beagle. We provide the parameters, methods, and packages used to train the SELR model in the Text File, available as supplementary data at *Bioinformatics* online, allowing users to retrain SELR with data from other phasing tools if needed. Additionally, users may choose to bypass SELR by directly inputting phased data or using a fixed phasing error rate, or as a latent variable which is marginalized in a Bayesian framework. This flexibility makes BEASTIE adaptable to a wide range of phasing methods, ensuring its utility across diverse datasets.

The flexibility of our Bayesian modeling framework offers numerous opportunities for future improvements. Given the widespread use of short-read sequencing and the high costs of long-read sequencing, our approach remains relevant. There is a substantial amount of legacy short-read data, and long-read data is often too expensive for extensive quantitative use. Combining legacy short-read data with Bayesian updating methods is thus advantageous. As long-read sequencing becomes more prevalent, integrating previous short-read datasets with new long-read data will be crucial, and Bayesian posteriors offer a principled method for this integration. Additionally, performing joint inference across all genes simultaneously, though computationally challenging, could provide significant benefits by borrowing strength across gene groups and pathways in a principled way. Future work could also apply our methods to other assays, such as ATAC-Seq, to explore allele-specific chromatin accessibility, or to combine information from transcriptomic and chromatin data to identify coordinated changes indicative of regulatory mechanisms. Additionally, the Bayesian framework offers the possibility of incorporating gene-specific priors from cohorts of control individuals, a feature that could be incorporated in future versions of the model.

## Supplementary Material

btaf283_Supplementary_Data

## Data Availability

Raw RNA-seq FASTQ files and VCF files used in this study are listed in Supplementary Text 1: Data Source. All model formulas, R code, and parameter configurations are provided in Supplementary Text 4: Calculation and Code, with model construction details in Supplementary Text 2: Helper Models, and benchmarking results in Supplementary Text 3: Results. The full analysis pipeline is available on GitHub at https://github.com/x811zou/BEASTIE and archived on Zenodo at https://doi.org/10.5281/zenodo.15062124.
