# Supplementary material for: Bayesian estimation of allele-specific expression in the presence of phasing uncertainty
Source: Bioinformatics. 2025 May 6;41(6):btaf283. doi: 10.1093/bioinformatics/btaf283 (PMC12202007; doi:10.1093/bioinformatics/btaf283)
Supplement: btaf283_Supplementary_Data [file btaf283_supplementary_data.zip › Supplementary Tables Figures Revision.pdf]

Supplementary Tables

Methods	Paper	Alignment Bias Filter	Genotyping Error Filter
Single Site 2-sided binomial exact test (SNP level)	Degner et al., 2009 [26]	✓	
	Lappalainen et al., 2013 [27]	✓	
	Kukurba et al.,2014 [23]	✓	✓
	Romanel et al., 2015 (ASEQ) [20]		✓
Single Site 2-sided binomial exact test (gene level)	Rozowsky et al., 2011 (AlleleSeq) [36]	✓	
	Castel et al., 2015 (ASEReadCounter) [19]	✓	
	Castel et al., 2016 (PhASER) [16]	✓	
Pseudo phasing (gene level)	Mayba et al., 2014 (MBASED) [13]	✓	
Bayesian Model (gene level)	Skelly et al., 2011 [14]	✓	
	Harvest et al., 2014 (QuASAR) [22]		✓
	Deonovic et al., 2016 (IDP-ASE) [37]		✓
	BEASTIE	✓	✓

Table S1: ASE Quantification Methods Used in Previous Studies

Hypothetical Parents	EUR-GBR	EAS-CHS	AMR-PUR	AFR-ACB	SAS8-PJL
Percentage of Het SNPs can be Phased by Trio	80%	80%	81%	83%	81%

Table S2. Expected percentage of bi-allelic heterozygous SNPs that cannot be phased using trio-based phasing. Data in 1000 Genomes Project samples from five ancestries, based on transmission simulations.

Sample	Ancestry	Sex
HG00096	EUR-GBR	M
HG00097	EUR-GBR	F
HG00403	EAS-CHS	M
HG00404	EAS-CHS	F
HG00551	AMR-PUR	F
HG00553	AMR-PUR	M
HG02307	AFR-ACB	M
HG02308	AFR-ACB	F
HG03237	SAS-PJL	M
HG03238	SAS-PJL	F

Table S3: 1000 Genome sample Used in Trio Phasing Assessment from Table S2.

Sample	Phasing method	Ancestry	Average SHAPEIT2 Switching Error per SNP pair	Shapeit2 reference panel
NA12878	GIAB gold standard VCF phasing	CEU	4.03% (974 out of 24167 SNP pairs)	All reference panel after removing NA12878
NA12878	GIAB gold standard VCF phasing	CEU	3.02% (731 out of 24167 SNP pairs)	European reference panel after removing NA12878
GM19440	Experimental VCF phasing	YRI	4.03% (2081 out of 51700 SNP pairs)	African reference panel
NA19240	1000 Genome trio phasing (parents: NA19238, NA19239)	YRI	2.2% (755 out of 34278 SNP pairs)	African reference panel

Table S4. Phasing error for each dataset. This phasing error is calculated using all phasable SNP pairs.

	hets	total_genes	genes_with_errors	percentage_with_errors
1	1	3114	0	0.000000
2	2	1841	114	6.192287
3	3	1334	121	9.070465
4	4	919	100	10.881393
5	5	625	75	12.000000
6	6	460	60	13.043478
7	7	358	52	14.525140
8	8	258	53	20.542636
9	9	162	32	19.753086
10	10	140	22	15.714286
11	11	102	14	13.725490
12	12	62	15	24.193548
13	13	62	16	25.806452
14	14	36	11	30.555556
15	15	35	6	17.142857
16	16	16	3	18.750000
17	17	20	4	20.000000
18	18	9	0	0.000000
19	19	18	4	22.222222
20	20	11	3	27.272727
21	21	8	2	25.000000
22	22	5	0	0.000000
23	23	2	1	50.000000
24	24	1	0	0.000000
25	25	2	0	0.000000
26	26	1	1	100.000000
27	27	4	3	75.000000
28	28	1	0	0.000000
29	29	1	0	0.000000
30	30	1	0	0.000000
31	32	1	0	0.000000
32	33	2	0	0.000000
33	35	1	0	0.000000
34	36	1	1	100.000000
35	38	1	1	100.000000
36	43	1	0	0.000000
37	106	1	0	0.000000

Table S5 Number of hets within a gene increases with higher switching error in GIAB (NA12878). The spearman correlation coefficient between the number of hets and percentage of genes with phasing error is 0.6854692 with p value as 0.002388332 (binning genes with hets with >= 18 together and removing genes with only 1 het).

MAF_group	N_SNPs	Percentage_SNPs	Average_switching_error
<0.2%	305	1.349259	48.550725
0.2%-0.5%	302	1.335988	21.582734
0.5-1%	244	1.079407	16.379310
1-5%	1431	6.330458	7.957245
>5%	20123	89.020128	3.120681

Table S6 Switching error decreases with high MAF in GIAB (NA12878)

Log10(distance) group	N_SNPs	Percentage_SNPs	Average_switching_error
<1	9	0.0398142	0.000000
1-2	673	2.9772174	2.678571
2-3	6659	29.4580845	2.543648
3-4	4915	21.7429772	3.968903
>4	2925	12.9396151	8.367769

Table S7 Switching error increases with high inter-distance between SNP Pair in GIAB (NA12878)

Variables used in fitted model	coefficient
intercept	-4.1059315
Minimum MAF in SNP pair	-6.0251976
Difference in MAF between SNP pair	1.8274357
Log10(inter distance in SNP pair)	0.5546364
r2	-0.4825783
Minimum MAF in SNP pair × Log10(inter distance in SNP pair)	0.7363362
Minimum MAF in SNP pair × d	-1.8066884
Difference in MAF between SNP pair × r2	2.0147905
r2 × d	-0.6216824
Minimum MAF in SNP pair × Difference in MAF between SNP pair × Log10(inter distance in SNP pair)	1.5263432
Minimum MAF in SNP pair × Difference in MAF between SNP pair × d	-18.8610889
Minimum MAF in SNP pair × Difference in MAF between SNP pair × Log10(inter distance in SNP pair) × r2×d	0.8808806

Table S8: variables with non-zero coefficients in fitted model with regularization
We use R function cva.glmnet and family “binomial” with 10 fold cross validation for 10 times on full model in training dataset and calculate the average alpha 0.7163 as the best. And then we use R function cv.glmnet and family “binomial” with 10 fold cross validation and best alpha in training dataset again to find the best lambda of 0.000326700507361396. This fitted model is then applied to testing dataset for switching error prediction.

chrN	num_SNPs	percent_SNPs	average_switching_error
1	2192	9.696970	5.020921
2	1642	7.263880	3.052065
3	1218	5.388188	3.855721
4	987	4.366291	4.148784
5	1049	4.640566	5.825243
6	995	4.401681	4.097117
7	1036	4.583057	3.202329
8	851	3.764654	6.271777
9	761	3.366512	6.329114
10	929	4.109710	2.446982
11	1305	5.773059	3.665521
12	1287	5.693431	2.311436
13	368	1.627958	7.111111
14	783	3.463835	4.863813
15	793	3.508073	1.795332
16	1249	5.525326	2.863688
17	1383	6.118115	4.468085
18	523	2.313647	7.068063
19	1667	7.374475	4.967801
20	476	2.105729	2.816901
21	300	1.327140	1.951220
22	811	3.587702	4.166667

Table S9 Switching error for each chromosome in GIAB (NA12878)

A

d_group <fctr>	num_SNPs <int>	percent_SNPs <dbl>	average_switching_error <dbl>
<0.2	328	1.451006	20.743034
0.2–0.4	251	1.110374	19.678715
0.4–0.6	253	1.119221	11.067194
0.6–0.8	374	1.654501	8.086253
>0.8	13354	59.075426	2.999323

B

r2_group <fctr>	num_SNPs <int>	percent_SNPs <dbl>	average_switching_error <dbl>
<0.2	4741	20.973236	9.096692
0.2–0.4	1779	7.869940	2.317694
0.4–0.6	1263	5.587259	1.352426
0.6–0.8	1176	5.202389	1.794872
>0.8	5601	24.777704	1.163415

Table S10. Switching error rate decrease with high LD in GIAB (NA12878)

ASE rate per ind %#	quick BEAST	Naive Sum	Major Site
average count per het >= 30	10.00% (193)	9.10% (176)	6.32% (121)
average count per het >= 10	7.23% (271)	6.39% (240)	4.37% (163)
average count per het >= 1	4.26% (311)	3.34% (245)	2.12% (155)

Table S11: Average %(#) ASE genes detected by different methods among 445 individuals from 1000 Genome Project with different average count per het cutoff

n (95%CI)	Utah (CEU)	Finnish (FIN)	British (GBR)	Tuscan (TSI)	Yoruba (YRI)
# individual	89	92	86	91	87
RNA total sequencing depth	58085673	59294884	59908207	58410071	62235536
# genes	3430 (3354,3505)	3503 (3420, 3587)	3583 (3504, 3662)	3529 (3448, 3611)	4387 (4284,4489)
# het snps	10945 (10611,11280)	11294 (10922,11665)	11658 (11299,12016)	11381 (11038,11724)	15474 (14968,15980)
average # hets	3.18	3.21	3.24	3.21	3.51
# ASE genes	267 (251,283)	247 (233,261)	249 (236,269)	253 (236,269)	342 (314,370)
% ASE genes	7.68 (7.65,7.72)	6.95 (6.92,6.98)	6.86 (6.83,6.90)	7.03 (7.00,7.07)	7.64 (7.58,7.69)

Table S12: Summary of 1000 Genome Project Dataset Analysis

gene set<br>(number of genes in set | number of genes individuals have)	dosage sensitivity	gene symbol/description
imprinted genes<br>(11 | 4)	tolerant	PBL monoallelic imprinted genes: PEG10, IGF2, SNRPN, NDN [33], [38], and LCL imprinted genes: SGCE, KCNQ1, SNURF, ZNF597, ZNF331, L3MBTL [38], and their common imprinted genes GNAS [33], [38]. (one RNA gene is excluded from this study: IPW)
LoF tolerant genes<br>(330 | 82)	tolerant	genes with at least two different high-confidence Loss-of-Function (LoF) variants found in a homozygous state in at least one individual [39].
RVIS recessive genes<br>(817 | 325)	tolerant	genes associated with recessive diseases from Online Mendelian Inheritance in Man (OMIM) genes [40].
housekeeping genes<br>(9 | 6)	intolerant	GAPDH, ACT8, PSMD2, PSMB3, TCL1A, PTDSS1, AAMP, YES1, HMBS [41] (Several RNA genes are excluded from this study: 18S, 28sRNA)
ClinGen haploinsufficiency genes<br>(294 | 101)	intolerant	Genes with sufficient evidence for dosage pathogenicity from The National Institutes of Health-funded Clinical Genome Resource (ClinGen) [42].
OMIM haploinsufficiency genes<br>(108 | 48)	intolerant	genes associated with haploinsufficiency from Online Mendelian Inheritance in Man (OMIM) genes [40].
MGI essential geneset<br>(2454 | 1309)	intolerant	Genes where homozygous knockout in mice results in pre-, peri- or post-natal lethality [43].
Autosomal dominant (AD) genes<br>(1083 | 339)	intolerant	genes associated with autosomal dominance from Online Mendelian Inheritance in Man (OMIM) genes [44].
Stillbirth genes<br>(24 | 21)	intolerant	genes with pathogenic variants known to cause disorders in infants and adults [45].
Non specialized genes		genes that do not belong to any of the geneset above

Table S13: geneset description

All numbers of genes in the table above are reflecting the number of genes that have at least 10 read counts per het in at least two of the individuals per ancestry group.

Geneset	Mean (Variance) of ASE Rate	95% CI of ASE rate	Wilcoxon Sign Rank Test P values
All genes	6.89 (3.11)	(6.73 , 7.06)	/
Non-specialized geneset	7.00 (3.09)	(6.84 , 7.17)	/
Imprinted	63.8 (1027)	(60.7 , 66.9)	1.20099e-65
LoF tolerant	13.4 (35.8)	(12.8 , 14.0)	1.186255e-61
Housekeeping	21.6 (706)	(19.0 , 24.1)	4.91415e-16
Recessive	5.98 (7.29)	(5.73 , 6.23)	1.394209e-24
Clingen haploinsufficient	3.97 (9.83)	(3.68 , 4.26))	1.85044e-55
OMIM haploinsufficient	5.29 (25.6)	(4.82 , 5.76)	1.142098e-16
All haploinsufficient	4.02 (9.26)	(3.74 , 4.30)	1.01169e-56
MGI essential	6.21 (4.11)	(6.21 , 6.73)	6.690214e-35
Autosomal Dominance (AD)	6.47 (7.77)	(6.21 , 6.73)	5.334317e-08
Still birth	1.64 (20.1)	(1.22 , 2.06)	4.036315e-52

Table S14: ASE Rate Statistics and Wilcoxon Test P-values for Defined-Geneset genes. P-values from the Wilcoxon Signed Rank test are conducted between defined-geneset genes and non-specialized genes.

Geneset	Mean (Variance) of ASE Magnitude	95% CI of ASE Magnitude	Permutation Test P values
All genes	1.30 (1.29)	(1.29 , 1.30)	/
Non-specialized geneset	1.31 (1.33)	(1.30 , 1.32)	/
Imprinted	3.49 (1.86)	(3.38 , 3.60)	9.999e-05
LoF tolerant	1.18 (0.501)	(1.15 , 1.21)	9.999e-05
Housekeeping	0.526 (0.467)	(0.439 , 0.613)	9.999e-05
Recessive	1.11 (0.547)	(1.08 , 1.13)	9.999e-05
ClinGen haploinsufficient	1.23 (0.792)	(1.17 , 1.29)	0.05609439*
OMIM haploinsufficient	1.02 (0.516)	(0.961 , 1.09)	9.999e-05
All haploinsufficient	1.16 (0.804)	(1.10 , 1.21)	0.00019998
MGI essential	1.29 (1.36)	(1.28 , 1.31)	0.09719028*
Autosomal Dominance (AD)	1.06 (0.821)	(1.04 , 1.09)	9.999e-05
Still birth	1.39 (0.808)	(1.17 , 1.62)	0.5507449*

Table S15: ASE magnitude Statistics and Permutation Test P-values for Defined-Geneset ASE genes. Permutation Test is conducted between ASE genes from defined-genesets and non-specialized genes. * indicates non-signifiance.

Geneset	Mean (Variance) of ASE Magnitude	95% CI of ASE Magnitude
All genes	0.251 (0.0394)	(0.250 , 0.251)
Non-specialized geneset	0.252 (0.0398)	(0.252 , 0.253)
Imprinted	0.213 (0.0564)	(0.186 , 0.241)
LoF tolerant	0.265 (0.0421)	(0.262 , 0.269)
Housekeeping	0.249 (0.0357)	(0.236 , 0.261)
Recessive	0.250 (0.0397)	(0.248 , 0.252)
Clingen haploinsufficient	0.243 (0.0379)	(0.240 , 0.246)
OMIM haploinsufficient	0.241 (0.0384)	(0.236 , 0.245)
All haploinsufficient	0.238 (0.0374)	(0.236 , 0.241)
MGI essential	0.241 (0.0370)	(0.240 , 0.242)
Autosomal Dominance (AD)	0.241 (0.0384)	(0.239 , 0.242)
Still birth	0.251 (0.0374)	(0.245 , 0.257)

Table S16: ASE magnitude Statistics and Permutation Test P-values for Defined-Geneset non-ASE genes.

	gene_name	gene_ID	avg_hets	total	num_ASE	num_extremeASE	extremeASE_ratio	ASE_ratio
1	SNRPN	ENSG00000128739	1.124424	217	217	217	1.0000000	1.00000000
2	MTRNR2L12	ENSG00000269028	1.315068	146	146	146	1.0000000	1.00000000
3	PEG10	ENSG00000242265	2.276753	271	271	271	1.0000000	1.00000000
4	POTEJ	ENSG00000222038	1.000000	15	15	15	1.0000000	1.00000000
5	NAP1L5	ENSG00000177432	1.406593	91	91	91	1.0000000	1.00000000
6	EIF5AL1	ENSG00000253626	1.825000	40	40	40	1.0000000	1.00000000
7	HLA-DQA2	ENSG00000237541	1.697674	86	86	86	1.0000000	1.00000000
8	UBBP4	ENSG00000263563	1.000000	59	59	59	1.0000000	1.00000000
9	FAM50B	ENSG00000145945	1.141414	99	98	99	1.0000000	0.98989899
10	LPAR6	ENSG00000139679	1.051546	97	95	97	1.0000000	0.97938144
11	UTS2	ENSG00000049247	1.576923	26	25	26	1.0000000	0.96153846
12	IGHV3-9	ENSG00000211940	1.117647	34	32	34	1.0000000	0.94117647
13	IGHV3-20	ENSG00000211946	1.312500	48	45	48	1.0000000	0.93750000
14	FLVCR1-DT	ENSG00000198468	2.165714	175	146	174	0.9942857	0.83428571
15	GEMIN2	ENSG00000092208	1.969163	227	196	224	0.9867841	0.86343612
16	AC021317.19	ENSG00000174093	1.126697	221	216	218	0.9864253	0.97737557
17	ZDBF2	ENSG00000204186	4.637037	135	132	133	0.9851852	0.97777778
18	PDE4DIP	ENSG00000178104	9.114754	61	58	60	0.9836066	0.95081967
19	IGKV3-15	ENSG00000244437	1.162791	43	37	42	0.9767442	0.86046512
20	AP3S1	ENSG00000177879	1.936508	378	368	369	0.9761905	0.97354497
21	IGLV3-1	ENSG00000211673	1.225000	40	37	39	0.9750000	0.92500000
22	IGHV3-21	ENSG00000211947	1.145161	62	59	60	0.9677419	0.95161290
23	IGLC7	ENSG00000211685	1.032967	91	81	88	0.9670330	0.89010989
24	FAM153A	ENSG00000170074	3.035714	28	26	27	0.9642857	0.92857143
25	WASH4P	ENSG00000234769	1.178571	56	41	54	0.9642857	0.73214286

Table S17: Top 25 genes identified by 'prioritization method' statistics.

The ranking order is determined by using the ratio of individuals with extreme ASE and the ratio of individuals with ASE per gene. ASE status is determined by FDR adjusted p-values from quickBEAST with a significance threshold of 0.05. And each gene has at least 10 read depth per het. There are 3 pseudogenes (MTRNR2L12, UBBP4, WASH4P), 2 RNA genes (FLVCR1-DT, AC021317.19), 6 immune gene segments (IGHV3-9, IGHV3-20, IGKV3-15, IGLV3-1, IGHV3-21, IGLC7).

Gene name	Gene ID	n individual	Primary SNP (prevalence % among individuals, rsid, AF, VEP link)	fisher exact test I p-value on primary SNP (allele types VS ASE)
SNRPN	ENSG00000128739	217	Chr15:25219512 (92%) rs705	1.0
MTRNR2L12	ENSG00000269028	146	Chr3:96336236 (95%) rs7625370 AF: 0.7	1.0
PEG10	ENSG00000242265	271	Chr7:94296769 (51%) rs138576679	1.0
POTEJ	ENSG00000222038	15	Chr2: 131414669 (73%) rs541111504	1.0
NAP1L5	ENSG00000177432	91	Chr4:89617387 (81%) rs8605	0.49648
EIF5AL1	ENSG00000253626	40	Chr10: 81273490 (100%)	1.0
HLA-DQA2	ENSG00000237541	86	Chr6:32713030 (43%) rs3208181	1.0
UBBP4	ENSG00000263563	59	Chr17: 21731041 (98%) rs145087783	1.0
FAM50B	ENSG00000145945	99	Chr6:3851193 (93%) rs6597007	1.0
LPAR6	ENSG00000139679	97	Chr13:48987032 (68%) rs2227311	0.49231
UTS2	ENSG0000049247	26	Chr1: 7913430 (73%) rs2890565	1.0
IGHV3-9	ENSG00000211940	52	Chr14: 106552310 (29%) rs8020204 MAF: 0.13 Missense variant, high PolyPhen score https://grch37.ensembl.org/Homo_sapiens/Variation/Mappings?db=core;r=14:106551810-106552810;tl=W3YPq84Jm2Ilr6Je-10305573;v=rs8020204;vdb=variation;vf=825684838#ENST00000390600_825684838_A_tablePanel	0.6004

IGHV3-20	ENSG00000<br>211946	48	Chr14:106667592<br>(62%)	1.0
FLVCR1-<br>DT	ENSG00000<br>198468	175	Chr1:213031158<br>(99%)	1.0
GEMIN2	ENSG00000<br>092208	227	Chr14:39601190<br>(87%)	1.0
AC021317<br>.19	ENSG00000<br>174093	221	Chr17:36353761<br>rs62074778<br>(91%)<br>no information	1.0
ZDBF2	ENSG00000<br>204186	135	Chr2:207177327<br>(70%)	1.0
PDE4DIP	ENSG00000<br>178104	61	Chr1:144915624<br>(97%)	1.0
IGKV3-15	ENSG00000<br>244437	53	Chr2: 89384726<br>(30%) rs377598278	1.0
AP3S1	ENSG00000<br>177879	378	Chr5:115249271<br>(98.7%) rs3879477<br>AF: 0.494<br>https://grch37.ensembl.org/Homo_sapiens/Tools/VEP/Results?tl=qOFE5jLmTyHZaa0L-10305571	1.0
IGLV3-1	ENSG00000<br>211673	60	Chr22: 23223278<br>(12%) rs394321	0.48571
IGHV3-21	ENSG00000<br>211947	62	Chr14:106692143<br>(62.9%)	0.18025
IGLC7	ENSG00000<br>211685	91	Chr22:23264864<br>(61.5%)	0.57279
FAM153A	ENSG00000<br>170074	190	Chr5: 177140874<br>(55%) rs11738486	0.44063
WASH4P	ENSG00000<br>234769	56	Chr16:66640<br>(66.1%) rs368745239<br>MAF: 0.16<br>20% show NMD transcript variants<br>https://grch37.ensembl.org/Homo_sapiens/Tools/VEP/Results?tl=6RjwgHANhJJJiXyc-10305551	1.0

Table S18: Top 25 genes identified by 'prioritization method' fisher exact tests. Each SNP has to have at least 10 reads, and shared in at least 1 individual. 'n_individual' indicates the number of individuals having at least 10 reads on a given SNP.

Gene name	gene information from Genecards	prior evidence of imprinted expression
NAP1L5	This gene encodes a protein that shares sequence similarity to nucleosome assembly factors, but may be localized to the cytoplasm rather than the nucleus. This gene is located within a differentially methylated region (DMR) and is imprinted and paternally expressed.	• paternally expressed in all examined cattle tissues [46]
FAM50B	FAM50B (Family With Sequence Similarity 50 Member B) contains an intronless ORF that arose from ancestral retroposition. This gene is adjacent to a differentially methylated region (DMR) and is imprinted and paternally expressed in many tissues.	• human [47], [48]
LPAR6	The protein encoded by this gene belongs to the family of G-protein coupled receptors, that are preferentially activated by adenosine and uridine nucleotides	• human [48]
HLA-DQA2	This gene belongs to the HLA class II alpha chain family. The encoded protein forms a heterodimer with a class II beta chain. It is located in intracellular vesicles and plays a central role in the peptide loading of MHC class II molecules by helping to release the CLIP molecule from the peptide binding site.	• Genomic imprinting candidate gene on kidney data from TCGA [49]
ZDBF2	This gene encodes a protein containing DBF4-type zinc finger domains. This gene is imprinted and paternally expressed in lymphocytes but is more stochastically expressed in the placenta.	• Paternally expressed in parthenogenetic and normal embryos [50]<br>• Genomic imprinting candidate gene on kidney data from TCGA [49]
UTS2	This gene encodes a mature peptide that is an active cyclic heptapeptide absolutely conserved from lamprey to human. The active peptide acts as a vasoconstrictor and is expressed only in brain tissue. Despite the gene family name similarity, this gene is not homologous to urocortin, a member of the sauvagine/corticotropin-releasing factor/urotensin I family. Most of the proprotein is cleaved to make the mature peptide. Transcript variants encoding different preproprotein isoforms have been described for this gene.	• Placenta [51]

Table S19: 6 imprinted gene candidates reported in previous publication. There are among the top 25 genes from Table S16 arranged based on decreasing order of extreme ASE ratio, and ASE ratio. And all genes are having at least 10 read counts on average of each het.

	ASE present	ASE absent
REF allele (REF count > ALT count)	X1	X2
ALT allele (REF count <= ALT count)	X3	X4

Table S20: Two by two table examining the association between Allele Type and ASE status. The table displays the number of individuals with alleles that have higher count in genes with different ASE status. Fisher's Exact Test was applied to assess the statistical significance of this association in **Table S16**.

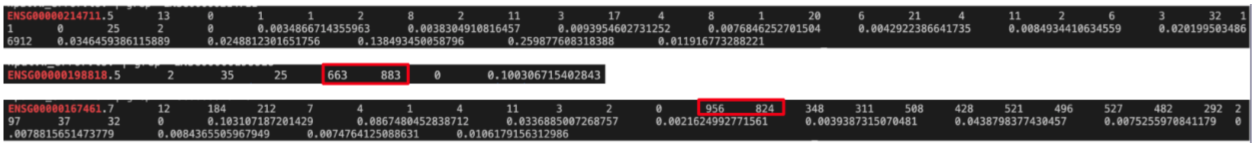

```
ENSG00000214711.5   13   0   1   1   2   8   2   11   3   17   4   8   1   20   6   21   4   11   2   6   3   32   1
1   0   25   2   0   0.0034866714355963   0.0038304910816457   0.0093954602731252   0.0076846252701504   0.0042922386641735   0.0084934410634559   0.020199503486
6912   0.0346459386115889   0.0248812301651756   0.138493450058796   0.259877608318388   0.011916773288221

ENSG00000198818.5   2   35   25   663   883   0   0.100306715402843

ENSG00000167461.7   12   184   212   7   4   1   4   11   3   2   0   956   824   348   311   508   428   521   496   527   482   292   2
97   37   32   0   0.103107187201429   0.0867480452838712   0.0336885007268757   0.0021624992771561   0.0039387315070481   0.0438798377430457   0.0075255970841179   0
.0078815651473779   0.0084365505967949   0.0074764125088631   0.0106179156312986
```

Table S21. Three genes in model input format used (in Supplementary Figure S15) to investigate the difference between fixing first site and fixing highest coverage site in qb.

(A) gene ENSG00000214711
(B) gene ENSG00000198818
(C) gene ENSG00000167461

π	θ	n	m	d
Rate of switching errors	Amount of ASE	Number of genes	Number of heterozygous site	Read depth per het site

Table S22: **Parameters in Parameterized Simulator.**

Variable	Definition
p	Probability of having major haplotype allele count at zero-count het SNP j
**α**	Sum of minor haplotype counts from all non zero-count sites at gene i
**β**	Sum of minor haplotype counts from all non zero-count sites at gene i
n	Total count at zero-count het SNP j
X	Predicted major haplotype allele count at zero-count het SNP j
var	Variance of total count from all het SNPs genomewide
mu	Mean of total count from all het SNPs genomewide

Table S23. Definitions of the variables in GEM model.

A

Test	total count: 100 allele frequency: 0.5	total count: 100 allele frequency: 0.9	total count: 100 allele frequency: 0.1
Fisher Type1	0.088	0.162	0.021
Betabino Type1	0.125	0.162	0.057
New model Type1	0.061	0.019	0.032
JAGS Type1	0.036	0.019	0.049
Fisher Power	0.945	0.99	0.276
Betabino Power	0.954	0.99	0.398
New model Power	0.935	0.98	0.323
JAGS Power	0.908	0.979	0.416

B

Test	total count: 5 allele frequency: 0.5	total count: 5 allele frequency: 0.9	total count: 5 allele frequency: 0.1
Fisher Type1	0.039	0.003	0.064
Betabino Type1	0.086	0.012	0.255
New model Type1	0.007	0.0	0.037
JAGS Type1	0.002	0.0	0.039
Fisher Power	0.709	0.957	0.167
Betabino Power	0.883	0.975	0.675
New model Power	0.637	0.92	0.131
JAGS Power	0.65	0.859	0.405

Table S24. Type 1 Error/Power Comparison Among Three Different Models using 1000 Stimulated Genes with 5% phasing error and 10 hets per gene. Fisher's Exact test and the beta binomial test do not control Type 1 Error, but that the GEM model does

	Power	Type 1 Error
BEASTIE	1.000	0.061
Naive Sum	0.921	0.046
Major Site	0.903	0.035
Pseudo Phasing	1.000	0.926

Table S25: Type 1 Error and power between qb, NS, MS and Pseudo Phasing. Type 1 error is using p values calculated from skewed t distribution fitted to 19M simulation data (calculation details in **Supplementary Text 4.1**) under the null with 5% phasing error and high coverage (10 hets per gene, read count 100 per het). The version of BEASTIE used here is quickBEAST.

Alpha	Observed Type 1 error	Expected Type 1 error	Absolute difference
5e-02	6.058631e-02	5e-02	1.058631e-02
5e-03	7.686211e-03	5e-03	2.686211e-03
5e-04	9.272225e-04	5e-04	4.272225e-04
5e-05	1.084970e-04	5e-05	5.849700e-05
5e-07	1.457733e-06	5e-07	9.577332e-07

Table S26: Observed Type 1 error and expected type 1 error with different alpha values in BEASTIE. Type 1 error is calculated using p values from skewed t distribution fitted to 19M simulation data ((calculation details in **Supplementary Text 4.1 (2)**) under the null with 5% phasing error and high coverage (10 hets per gene, read count 100 per het). The version of BEASTIE used here is quickBEAST.

Supplementary Figures

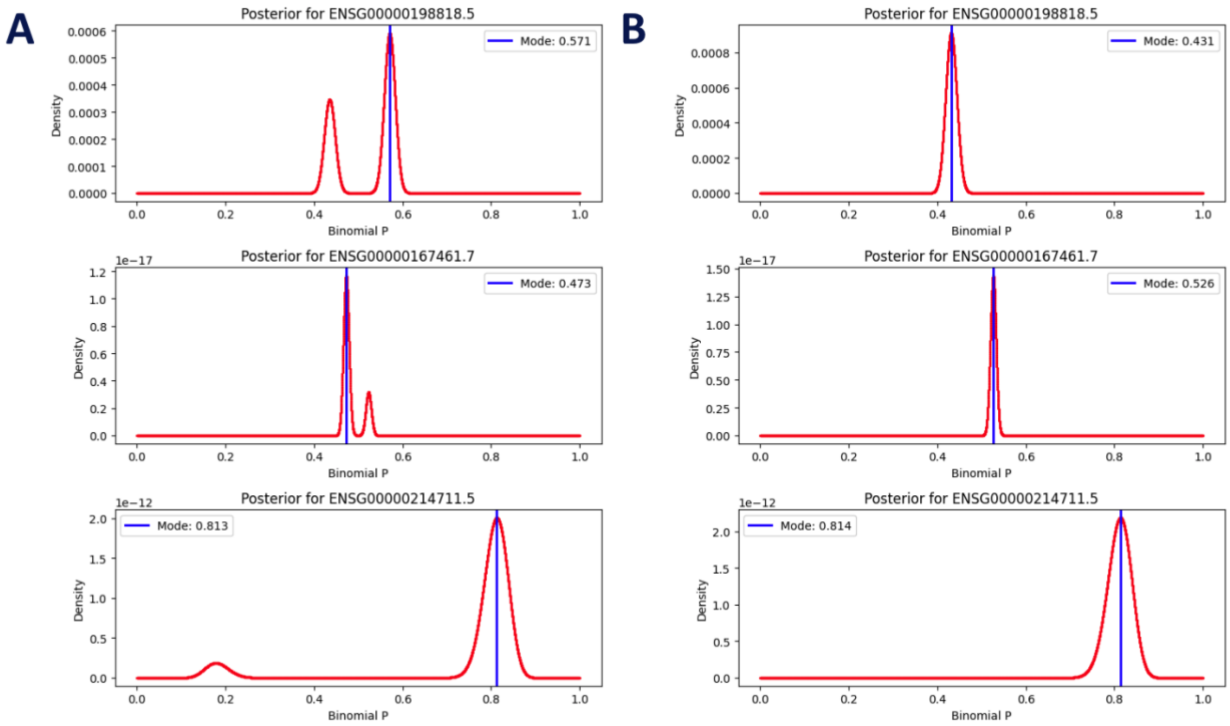

Figure S1. Fixing first site VS fixing highest coverage site in QuickBeast (qb) on three genes from NA12878 sample.

(A) qb with fixing the first site (multiple local maximum)

(B) qb with fixing the highest coverage site (single maxima)

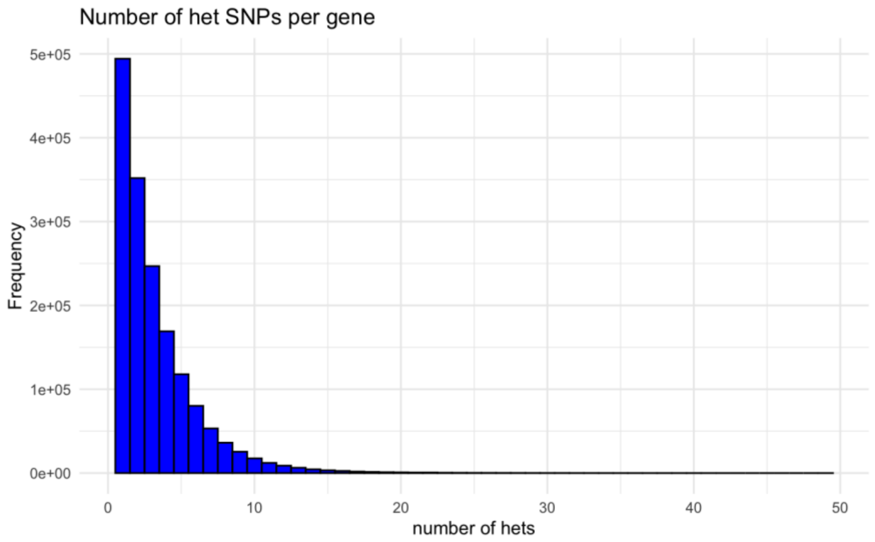

Figure S2. density plot of the number of exonic het SNPs per gene on 1000 genome samples.

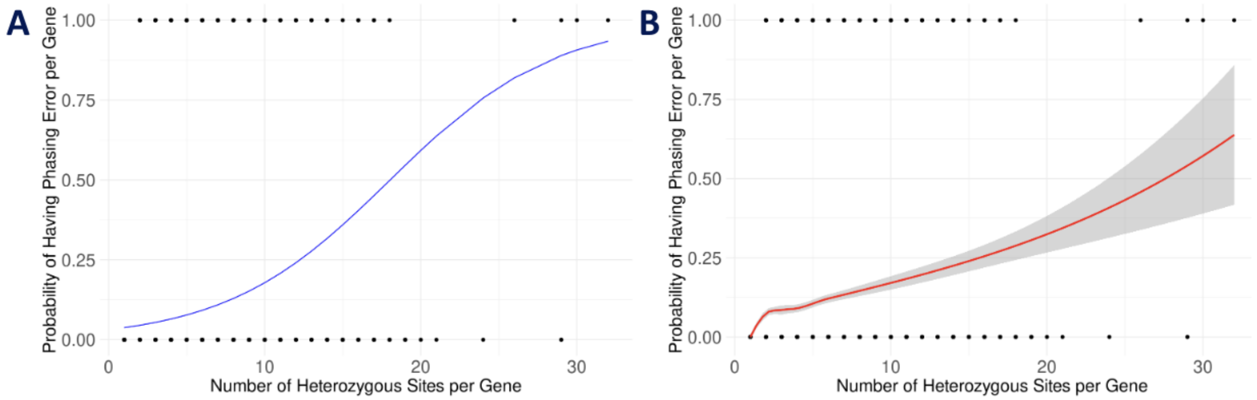

Figure S3. The relationship between the number of heterozygous site per gene and the probability of having phasing error based on gold standard phasing from GIAB data.

(A) Logistic Regression curve

(B) LOWESS curve

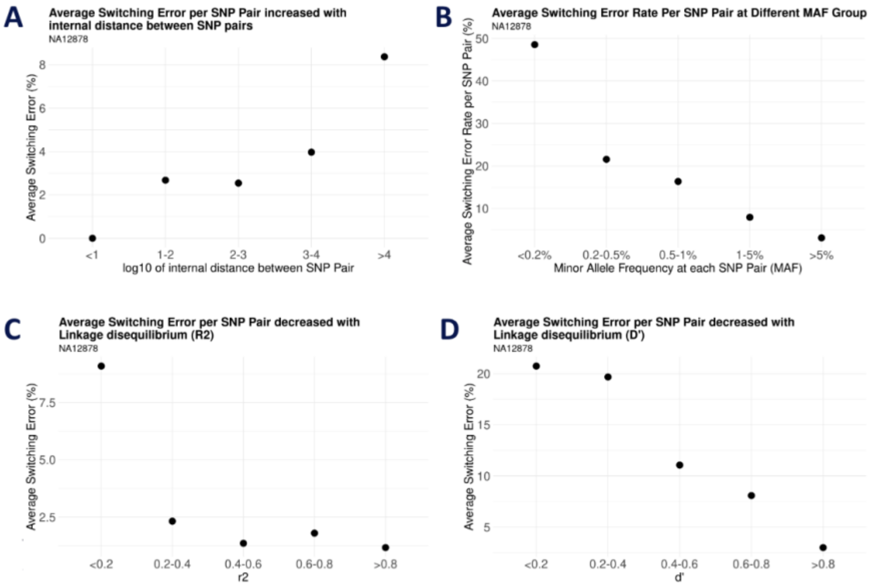

Figure S4. relationship of each variables (in SELR) with switching error

(A) Switching error increases with high inter-SNP pair distance
(B) Switching error decreases with high MAF
(C) Switching error decreases with high LD values (r2)
(D) Switching error decreases with high LD values (d')

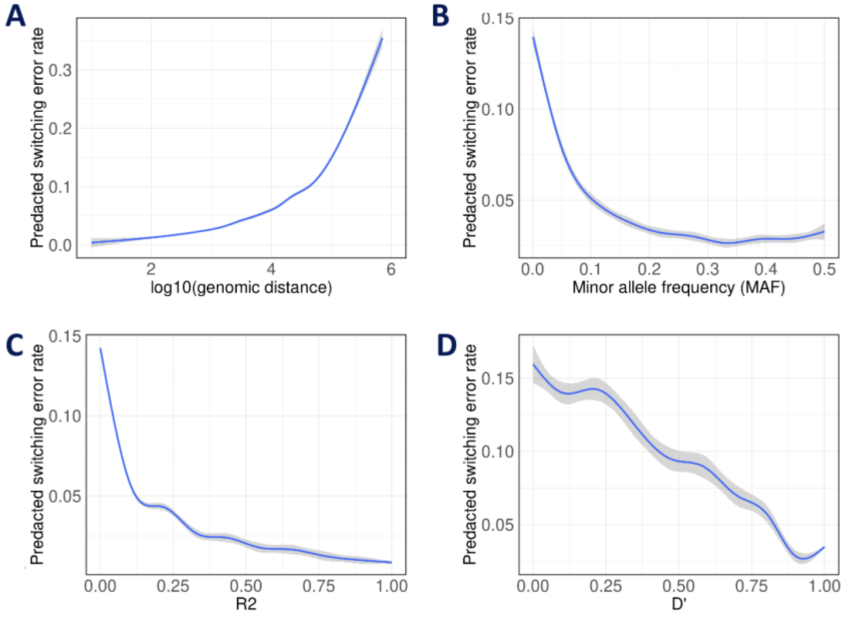

Figure S5. GAM smoothed line plot visualizing the relationship of each variables (in SWLR) with predicted switching error

(A) Predicted switching error increases with high inter-SNP pair distance between a SNP pair
(B) Predicted switching error decreases with high min_MAF (minimum MAF between a SNP pair)
(C) Predicted switching error decreases with high LD values (r2)
(D) Predicted switching error decreases with high LD values (d')

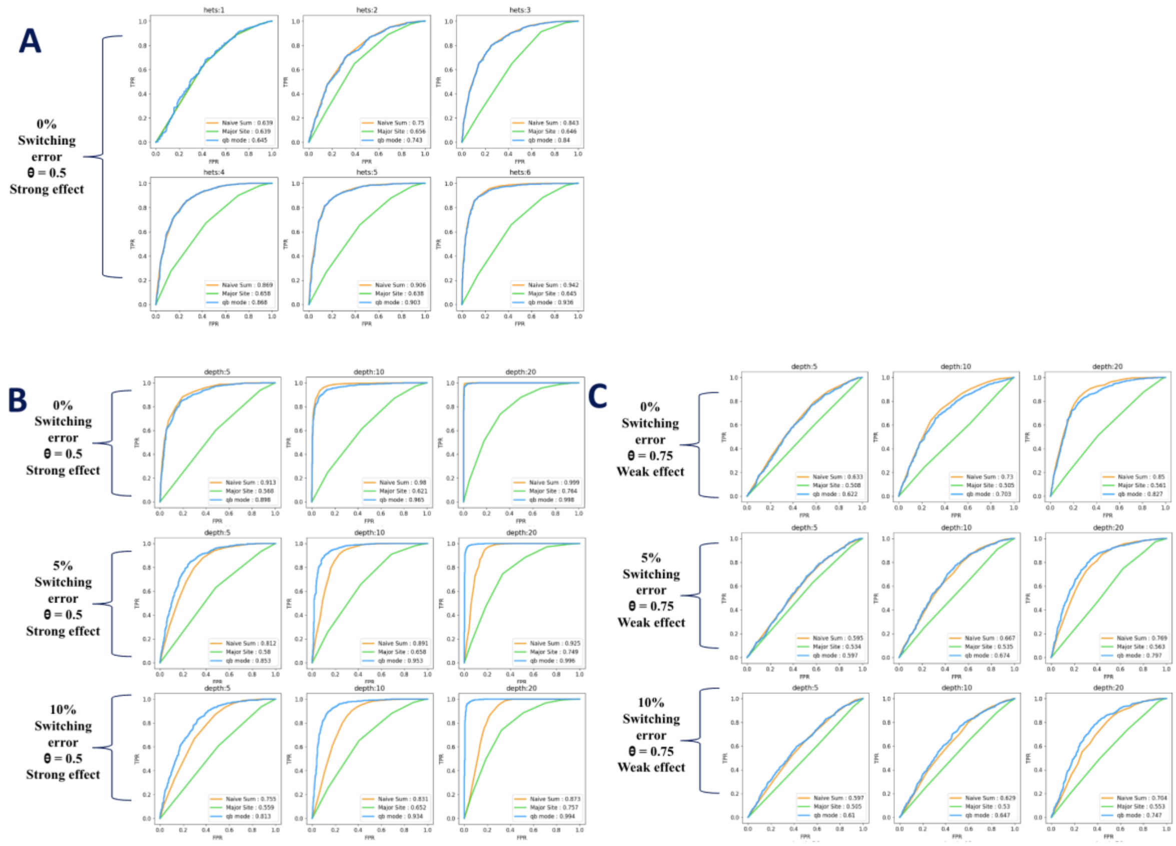

Figure S6. ROC curves for BEASTIE with default 5% phasing error and two baselines based on the binomial test (NaïveSum: naively summing across sites; MajorSite: using the site with highest total count) using simulated data by parametrized simulator. All results were generated using an optimized version of BEASTIE described in Supplementary Text Section 2.2.

a.ROC curves on simulated data with 0% switching error rate, 10 reads per site, for 1,2,3,4,5,6 heterozygous sites per gene , at strong ASE effect (θ=0.5).

b.ROC curves on simulated data with 0%, 5%, 10% switching error rate, 10 heterozygous sites per gene, for 5,10,20 reads per site , at strong ASE effect (θ=0.5).

c.ROC curves on simulated data with 0%, 5%, 10% switching error rate, 10 heterozygous sites per gene, for 5,10,20 reads per site, at weak ASE effect (θ=0.75).

All panels: numbers in legend are AUC values. Simulated data have 1000 genes.

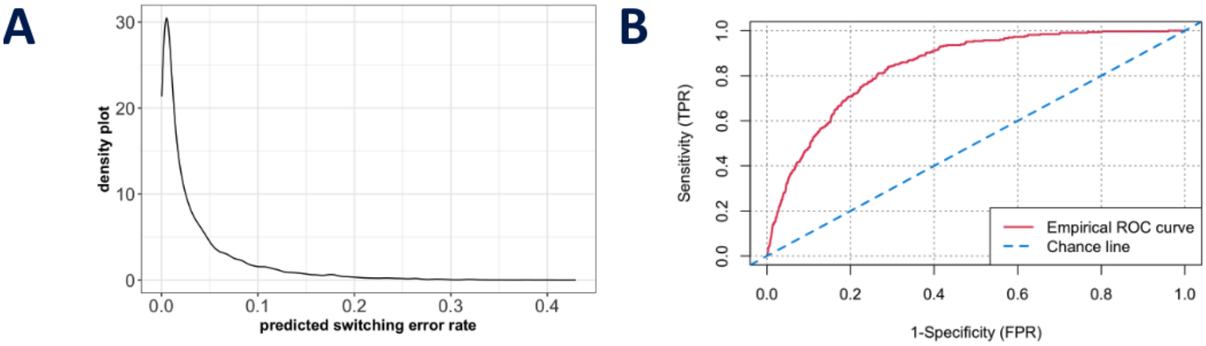

Figure S7. SELR model performance

(A) Scatterplot plot of predicted SHAPEIT2 switching error rate (by SELR) on sample GIAB (NA12878) as evaluated against GIAB gold standard phasing.

(B) ROC curve for classification of genes having ASE in test dataset (AUC = 0.8425). The model was trained on 8,729 site pairs and validated against a disjoint set of 8,730 site pairs, each set comprising approximately 3.7% incorrectly phased site pairs.

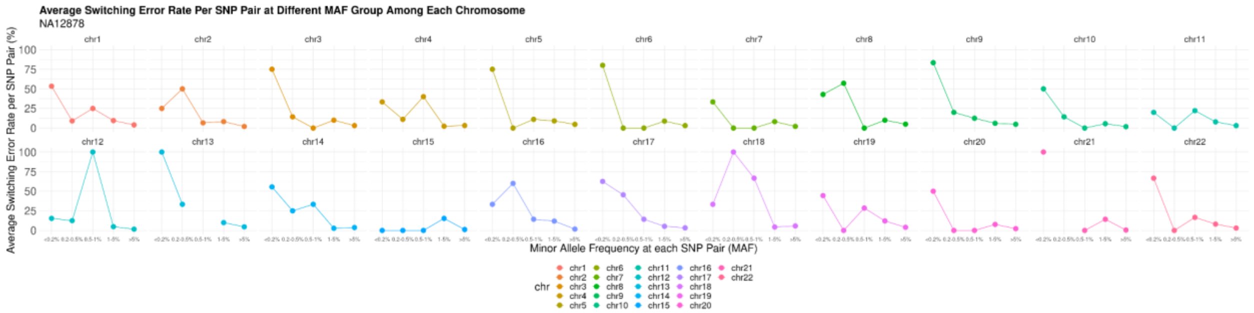

Figure S8 Switching error decreases with high MAF for each chromosome

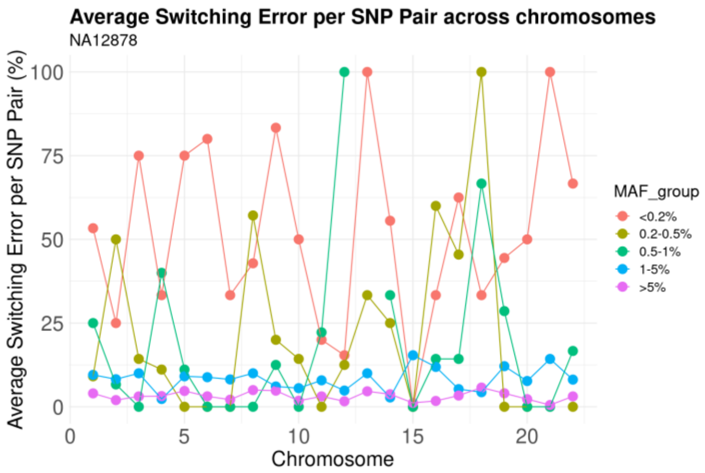

Figure S9 Switching error decreases with high MAF across chromosomes

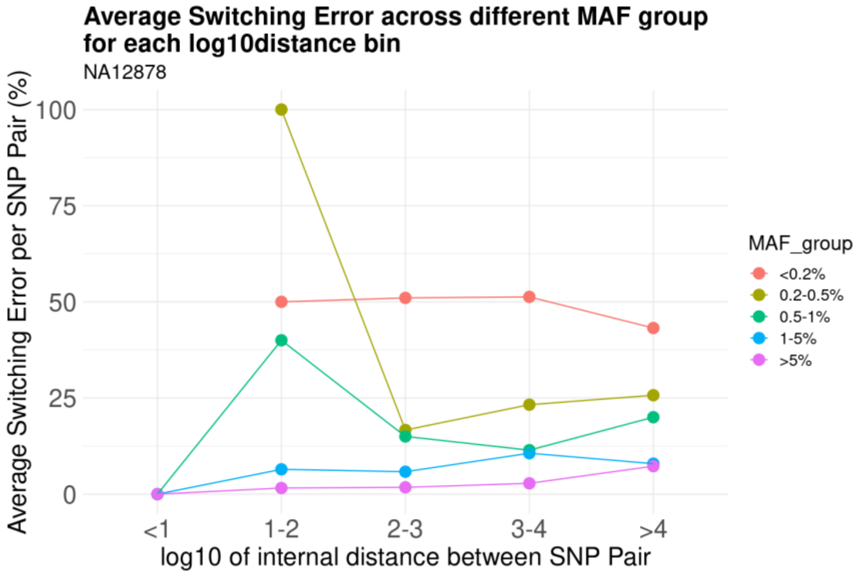

Figure S10 Switching error decreases with high MAF across inter-distance per SNP Pair

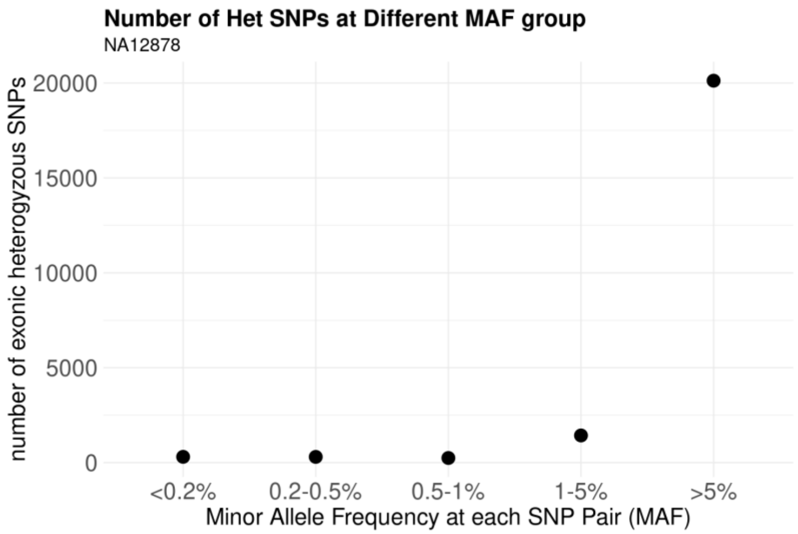

Figure S11 number of SNPs increases with high MAF

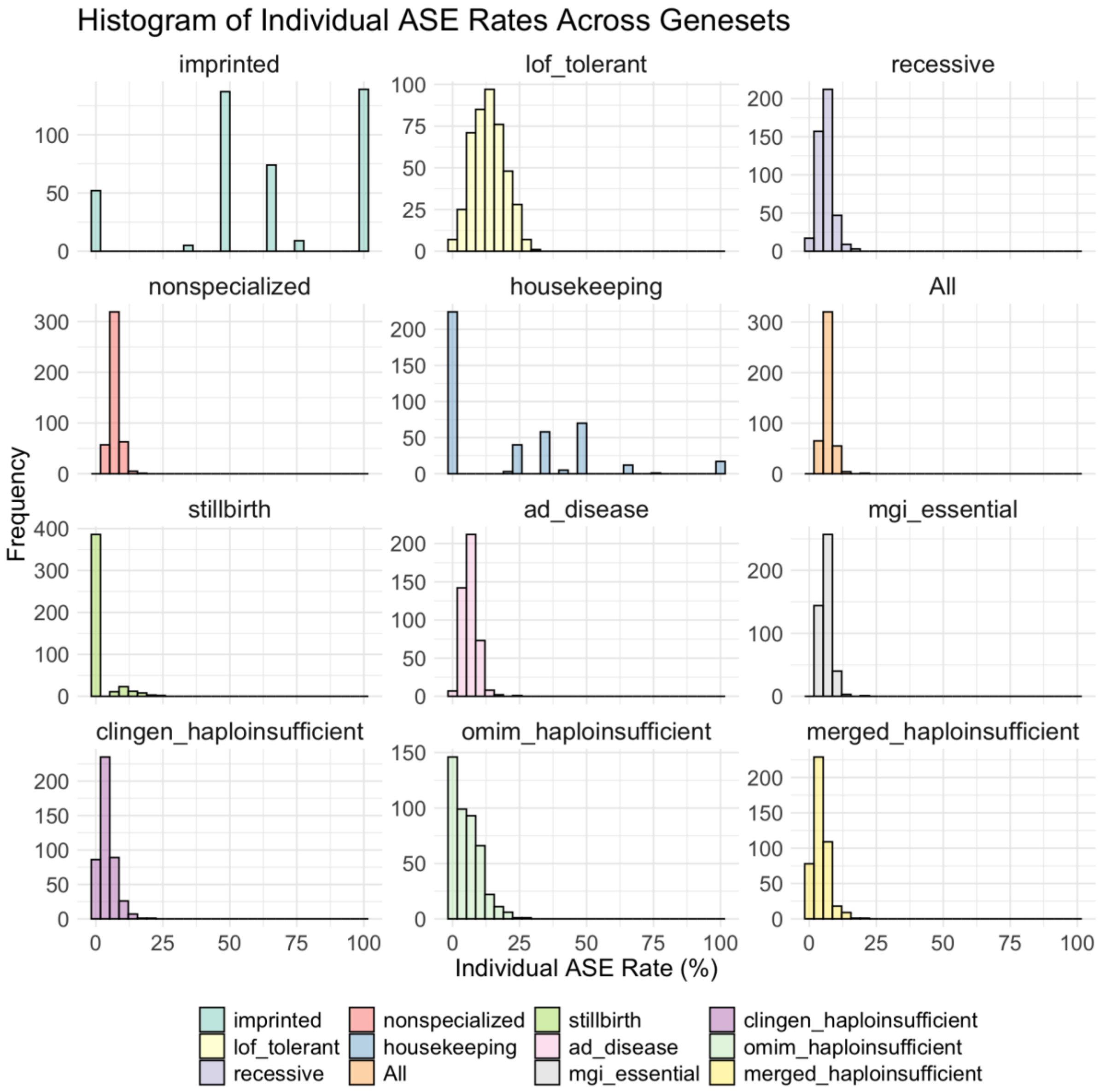

Figure S12: ASE Rate Histograms for all genes from different genesets

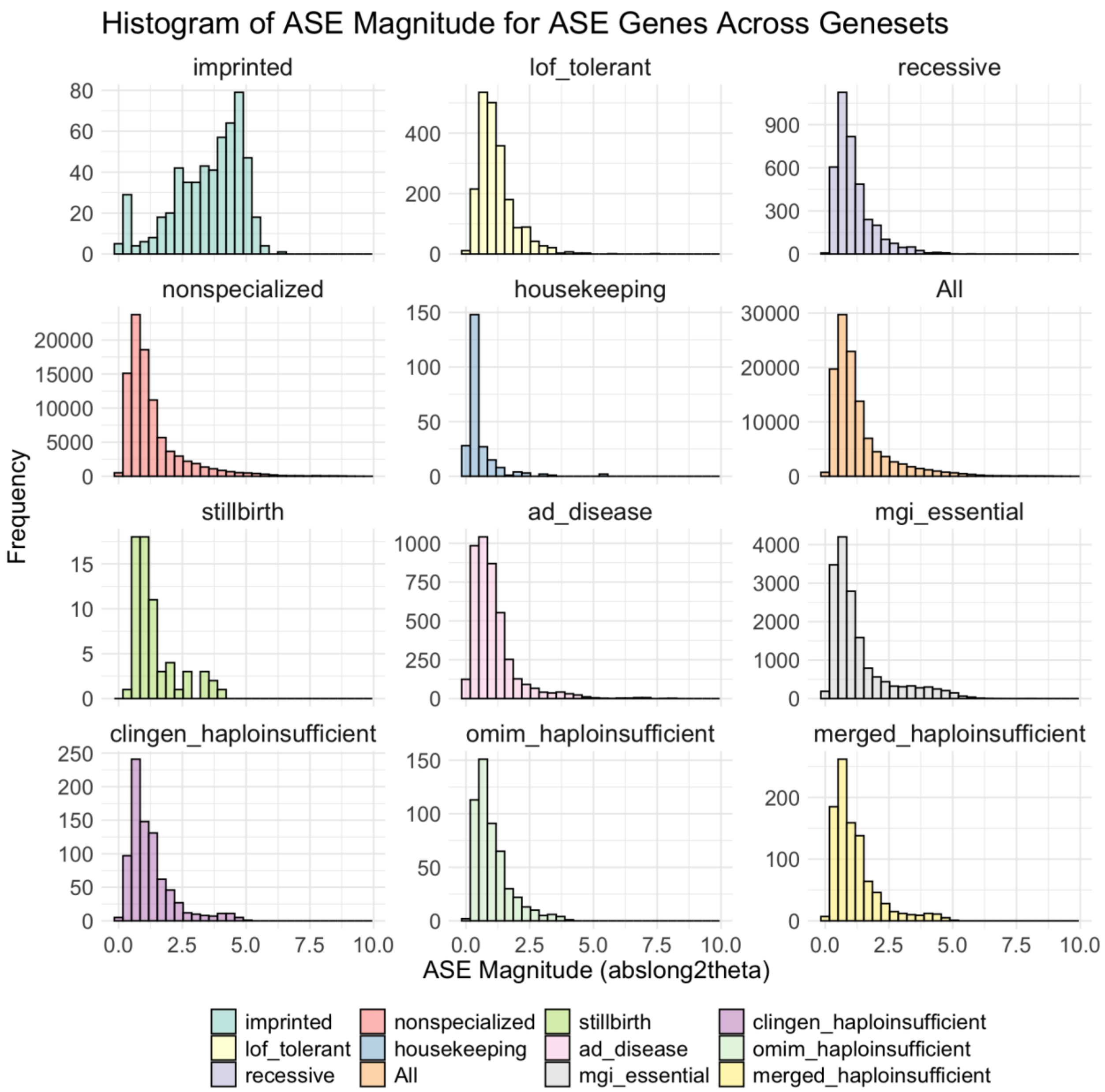

Figure S13: ASE magnitude histograms in ASE genes from different genesets

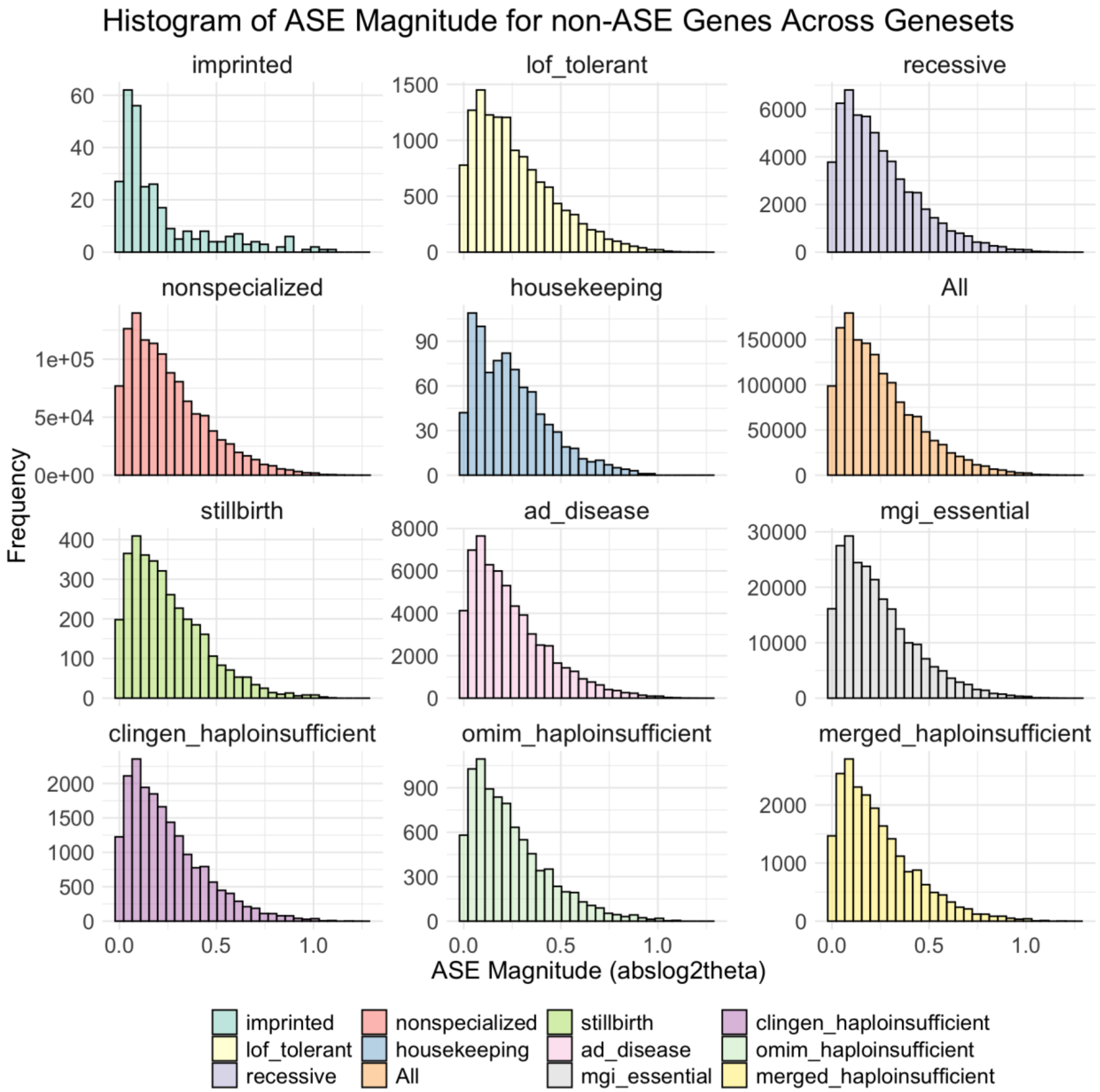

Figure S14: ASE magnitude histograms in non-ASE genes from different genesets

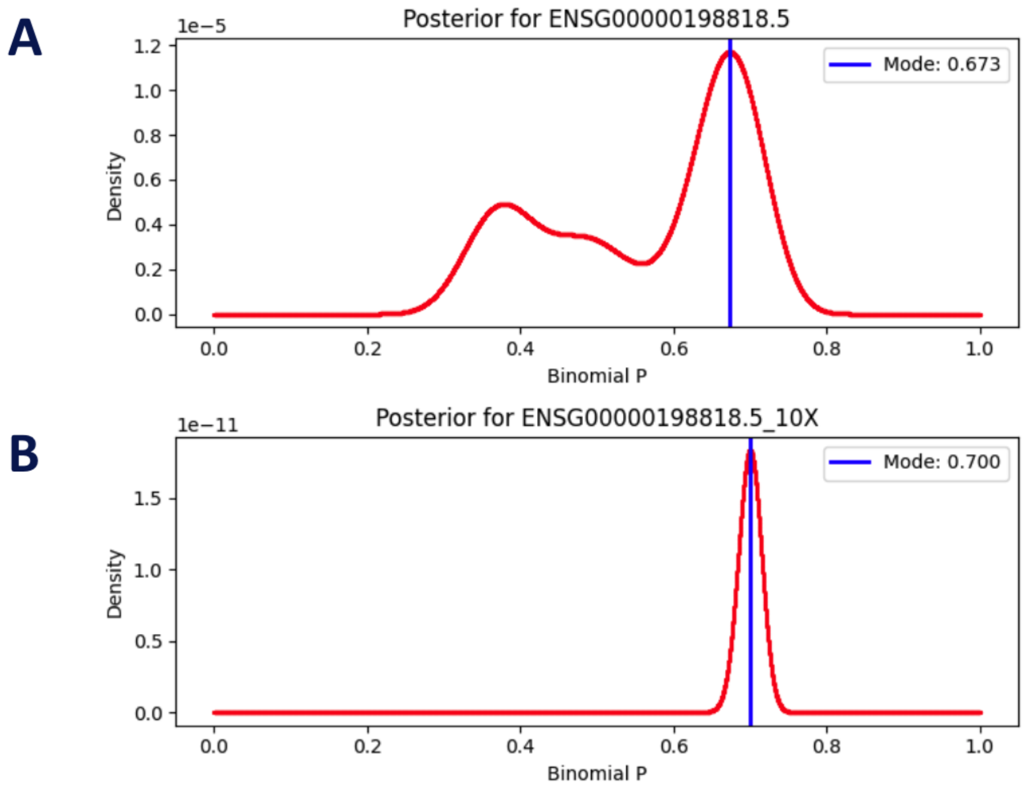

Figure S15. Trimodal distribution changes to Unimodel distribution after 10X coverage increases from NA12878 sample data of gene ENSG00000198818.5

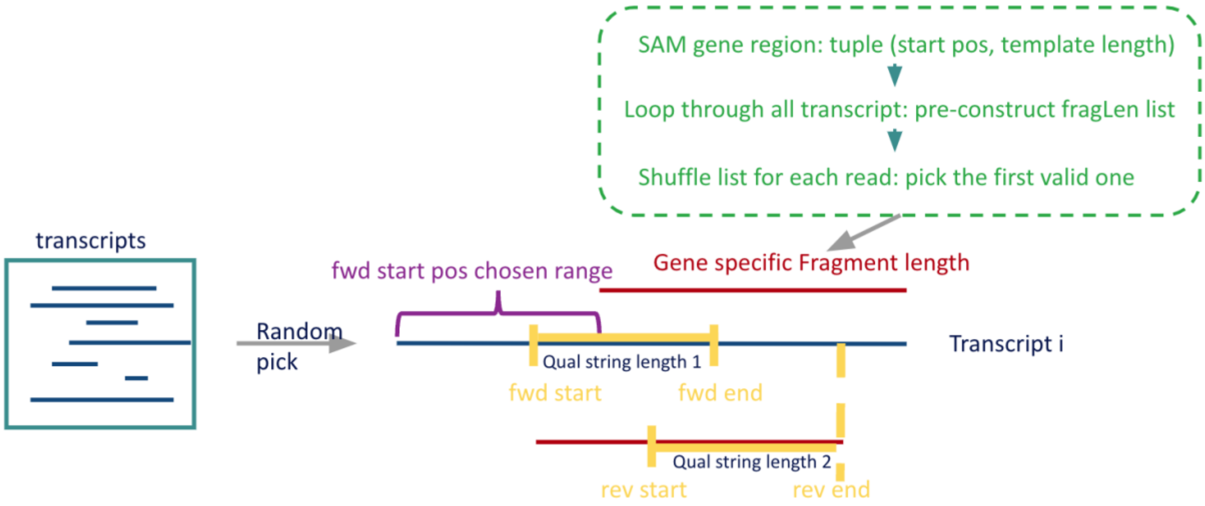

Figure S16. Diagram for unbiased splice reads simulator

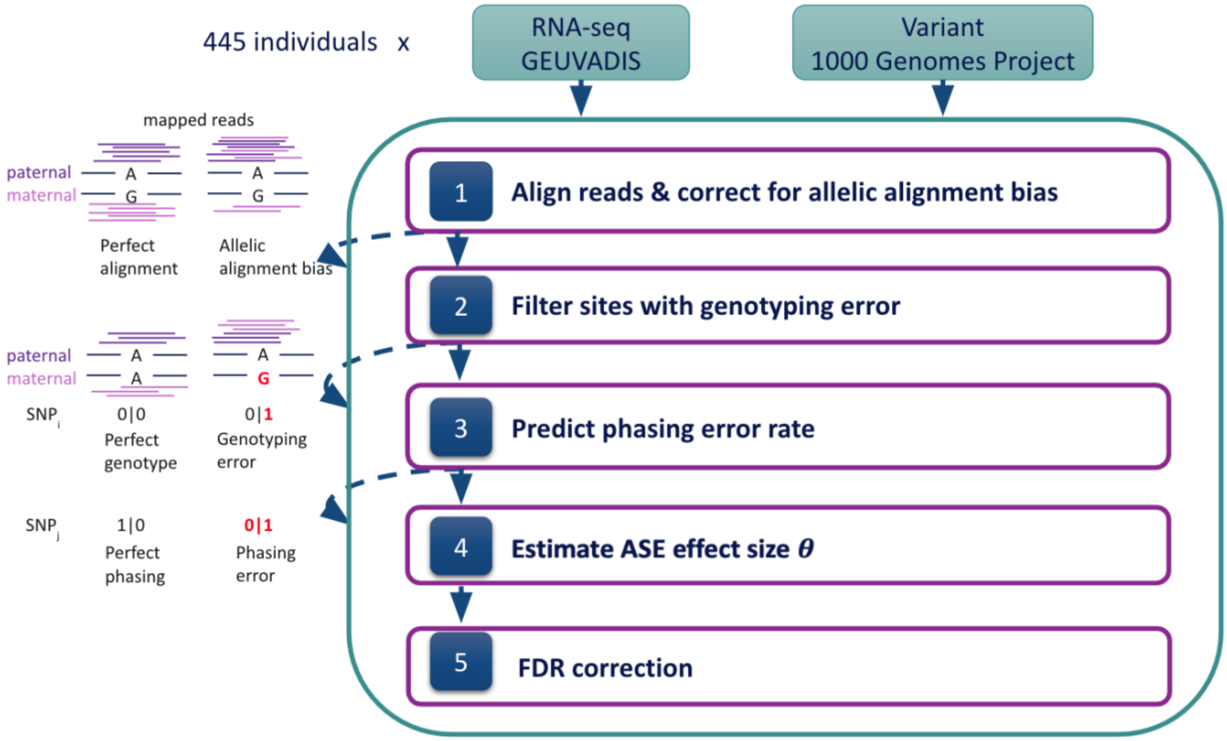

Figure S17: **pipeline overview.** It contains all major steps processing the steps that are explained in Methods.

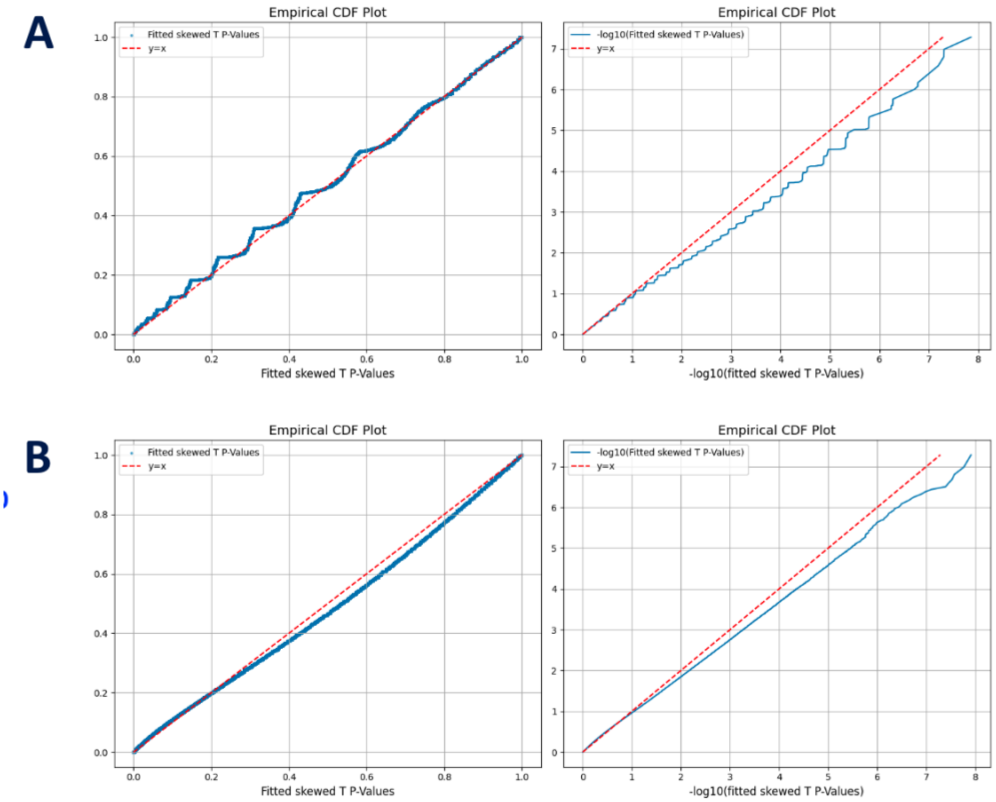

Figure S18: Empirical CDF using p values from 19M null simulations fit to skewed t distribution (calculation details in (**Supplementary Text 4.1 (2)**))

A: ECDF of fitted skewed t distribution p values and -log10 (p values) from 19207904 null stimulated genes with 3 hets and 30 read depth per het and theta = 1

B: ECDF of fitted skewed t distribution p values and -log10 (p values) from 19207904 null stimulated genes with 10 hets and 100 read depth per het and theta = 1

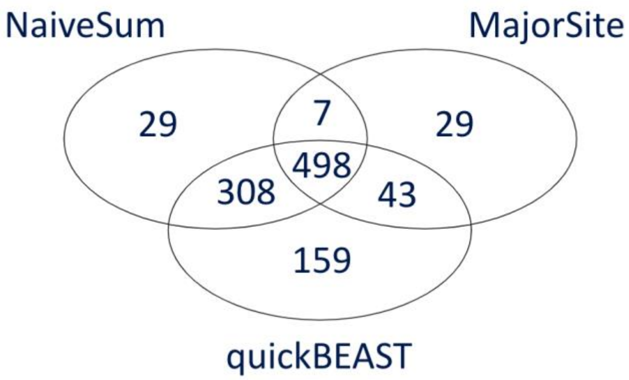

Figure S19: ASE results and comparative analysis on NA12878. Comparison with NaiveSum and MajorSite. Concordance of ASE genes detection is shown between BEASTIE, NaiveSum and MajorSite. The version of BEASTIE used here is the latest version called quickBEAST.

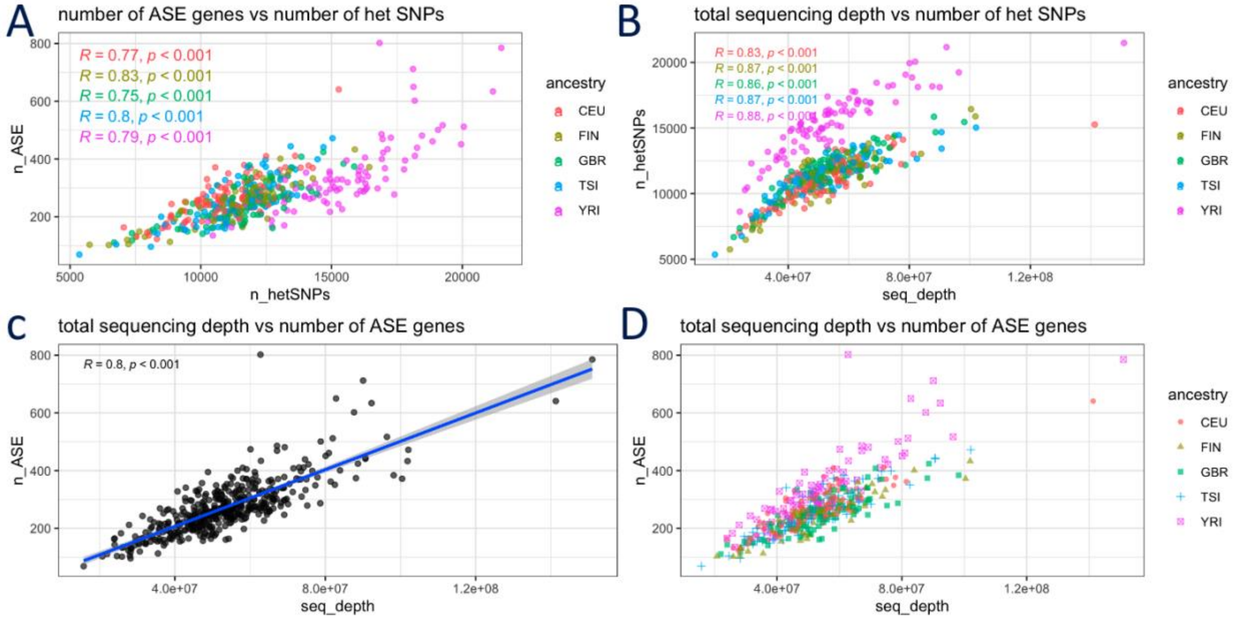

Figure S20. Positive pearson correlation between the total number of reads and ASE genes
(A)Scatterplot of the number of total heterozygous SNPs versus the number of ASE genes per individual.
(B)Scatterplot of the total number of reads from aligned BAM files versus the number of heterozygous sites per individual.
(C)Scatterplot of the total number of reads from aligned BAM files versus the number of ASE genes per individual.
All panels: legend indicates the color/shape for each ancestry. Pearson correlation coefficient and p-value are labeled in the plot.

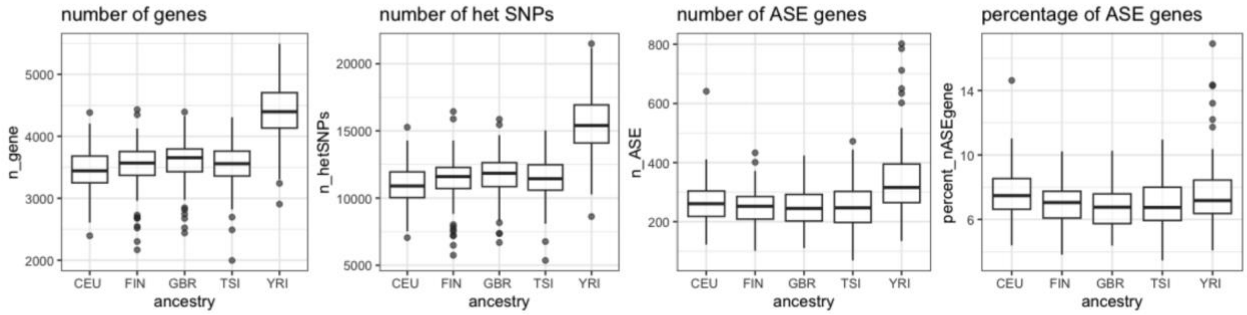

Figure S21: ancestry characteristics

Boxplot of number of genes, number of het SNPs, number of ASE genes, and percentage of ASE genes.

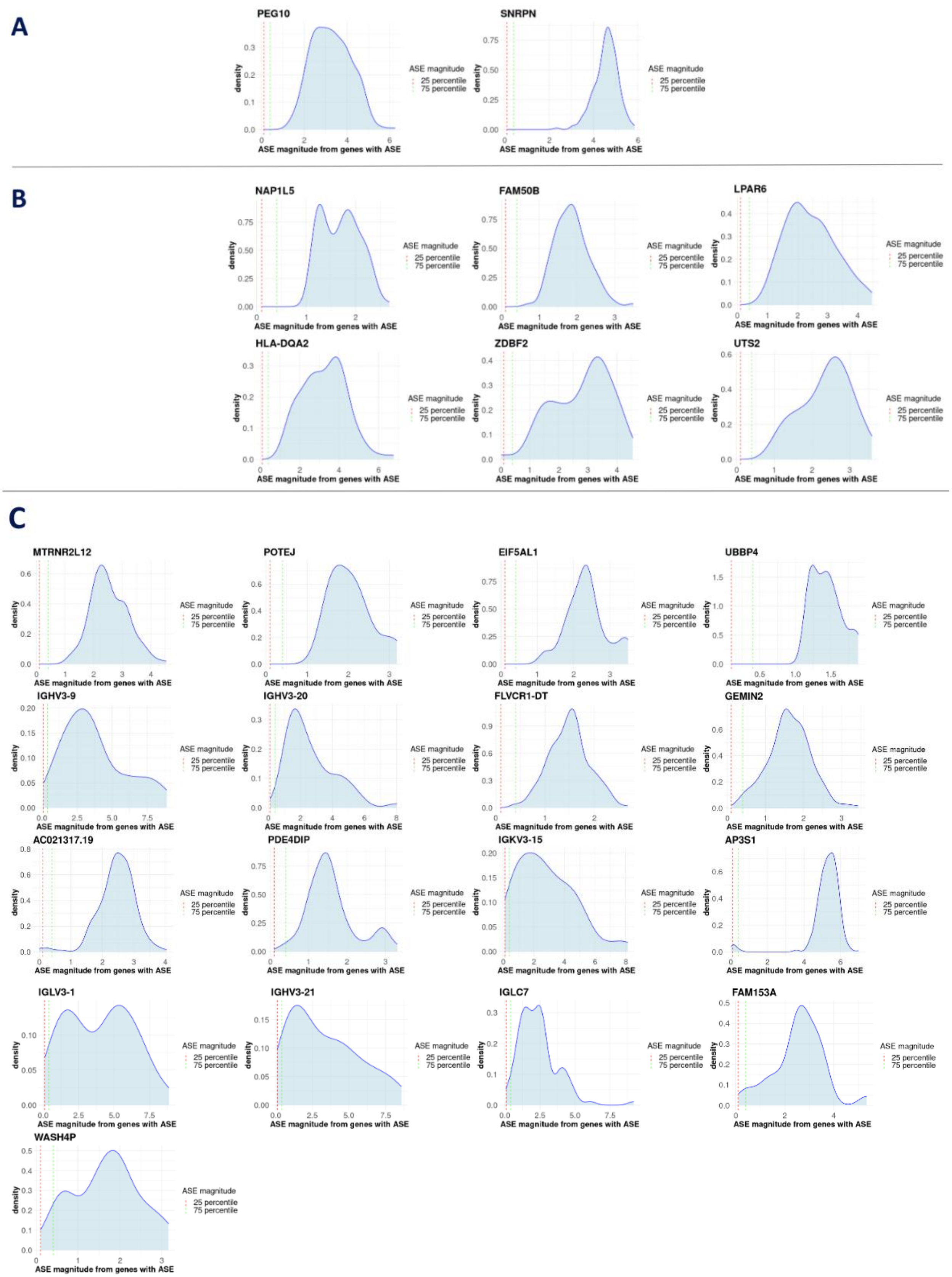

Figure S22. Density plot of ASE magnitude for (A) 2 known imprinted genes, (B) 6 previously

reported genes with imprinting status in specific tissues, (C) 17 genes with extreme ASE magnitude without prior literature on imprinting status.

A

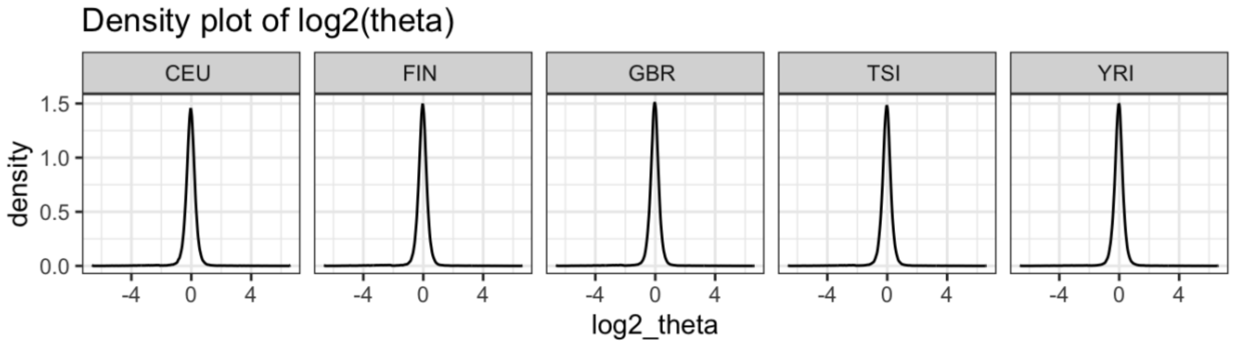

B

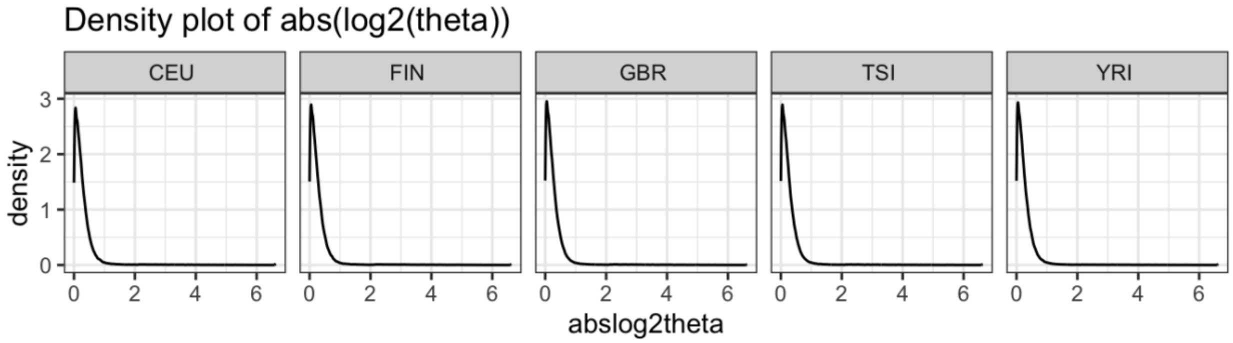

C

**ancestry** <chr>	**min_log2theta** <dbl>	**max_log2theta** <dbl>
CEU	−6.629357	6.629358
FIN	−6.629357	6.629367
GBR	−6.629357	6.629358
TSI	−6.629357	6.629358
YRI	−6.629357	6.629358

Figure S23. (A) density plot of log2(theta) among 5 ancestries, (B) density plot of abs(log2(theta) among 5 ancestries, (C) statistics of log2(theta) among 5 ancestries.

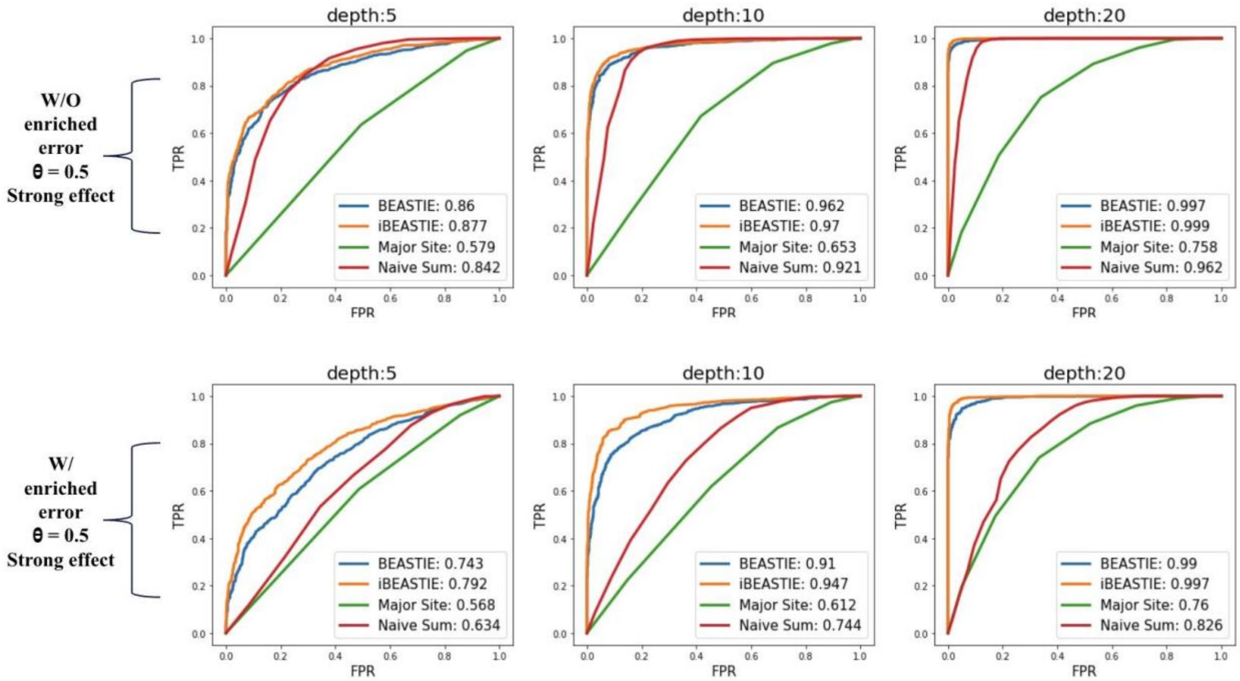

Figure S24. ROC Curves for BEASTIE and iBEASTIE on Simulated Data. BEASTIE with default phasing error parameter, IBEASTIE with predicted phasing error predicted from SELR.
(Upper) ROC curves on simulated data from GIAB data with 3.8% switching error, 10 heterozygous sites per gene, and coverage of 5, 10, or 20 reads per site, with strong ASE effect (θ = 0.5).
(Bottom) ROC curves on simulated data from GIAB data with 12% switching error (an odd number of switching errors per gene), 10 heterozygous sites per gene, and coverage of 5, 10, or 20 reads per site, with strong ASE effect (θ = 0.5).
All panels: Legends show AUC values. Semi-empirical simulators composed 1,000 genes with 10 heterozygous sites per gene from real GIAB data. ROC curves compare BEASTIE with predicted phasing error and two baseline methods. Results are from the BEASTIE stan version.
